# Membrane vesicles released by *Lacticaseibacillus casei* BL23 inhibit the biofilm formation of *Salmonella* Enteritidis

**DOI:** 10.1038/s41598-023-27959-9

**Published:** 2023-01-20

**Authors:** David da Silva Barreira, Julie Laurent, Jessica Lourenço, Julia Novion Ducassou, Yohann Couté, Jean Guzzo, Aurélie Rieu

**Affiliations:** 1 Université de Bourgogne Franche-Comté (UBFC), AgroSup Dijon, UMR PAM A 02.102, 21000 Dijon, France; 2 Univ. Grenoble Alpes, INSERM, CEA, UMR BioSanté U1292, CNRS, CEA, R2048, 38000 Grenoble, France

**Keywords:** Biotechnology, Microbiology

## Abstract

Biofilms represent a major concern in the food industry and healthcare. The use of probiotic bacteria and their derivatives as an alternative to conventional treatments to fight biofilm development is a promising option that has provided convincing results in the last decades. Recently, membrane vesicles (MVs) produced by probiotics have generated considerable interest due to the diversity of roles they have been associated with. However, the antimicrobial activity of probiotic MVs remains to be studied. In this work, we showed that membrane vesicles produced by *Lacticaseibacillus casei* BL23 (LC-MVs) exhibited strong antibiofilm activity against *Salmonella enterica* serovar Enteritidis (*S*. Enteritidis) without affecting bacterial growth. Furthermore, we found that LC-MVs affected the early stages of *S.* Enteritidis biofilm development and prevented attachment of bacteria to polystyrene surfaces. Importantly, LC-MVs did not impact the biomass of already established biofilms. We also demonstrated that the antibiofilm activity depended on the proteins associated with the LC-MV fraction. Finally, two peptidoglycan hydrolases (PGHs) were found to be associated with the antibiofilm activity of LC-MVs. Overall, this work allowed to identify the antibiofilm properties of LC-MVs and paved the way for the use of probiotic MVs against the development of negative biofilms.

## Introduction

The biofilm mode of life is predominant in nature and is characterized by the emergence of specific physiological properties compared with single planktonic cells^1^. Biofilms are defined as spatially organized communities of microorganisms embedded in a self-produced matrix of extracellular polymeric substances (EPS) that adhere to each other and/or an interface^2,3^. The matrix is a major structural element that contributes to the mechanical stability of biofilms, and the adhesion and immobilization of cells^4^. It contains a diversity of elements including polysaccharides, proteins, nucleic acids, lipids and membrane vesicles (MVs) (also referred to as extracellular vesicles). Both the matrix and cell activities lead to the development of physiochemical gradients (nutrient level, oxygen, redox, pH) within biofilms which result in cellular heterogeneity.

This heterogeneity and the protection provided by biofilms are key factors explaining the high resistance of bacteria to a variety of stresses, such as ultraviolet light^5–7^, phages^8^, desiccation^7^, temperature^9^, host immune systems^10,11^ and antibiotics^12,13^. Notably, it has been reported that bacterial biofilms were up to 1000 times more resistant to antibiotic treatments than planktonic cells^14^.

The high resistance of biofilms to antibiotics is a major issue in healthcare as it makes treatments difficult and promotes the emergence of antimicrobial resistances^15–17^. It is estimated that biofilms are involved in approximately 80% of chronic human infections^18^ and over 90% of chronic wounds^19^. They are found in the human body and in medical devices such as catheters, pacemakers, endotracheal tubes, and prosthetic implants^20,21^. Biofilms are also a challenge in agri-food industries where they contaminate food, crops and develop on industrial infrastructures such as equipment and water pipelines^22,23^.

*Salmonella enterica* subsp. *enterica* is a major foodborne pathogen which caused 93.8 million gastroenteritis cases worldwide in 2006, resulting in 155 000 deaths^24^. The serovar Enteritidis (*S.* Enteritidis) is the most common serotype and infects humans through contaminated food such as water, meat, poultry, vegetables, and fruits. *S.* Enteritidis poses considerable difficulties in the food industry since it can form biofilms on diverse surfaces and is often found on eggs and chicken meat.

To address the rise of resistance and the failure of conventional treatments to eliminate biofilms in healthcare and the food industry, new alternatives must be investigated. Probiotics have been found to be effective against biofilms of various *Salmonella* strains^25–27^ as well as other pathogens^28–32^.

Probiotics are defined by the World Health Organization as “live microorganisms which, when administered in adequate amounts, confer a health benefit on the host”^33^. A large number of probiotic bacteria used in the food industry are members of the diverse *Lactobacillaceae* family and are “Generally Regarded as Safe” (GRAS). Lactobacilli are rod shaped Gram-positive bacteria found in a variety of ecological niches including the human gastrointestinal tract. Several members of the *Lacticasibacillus casei* species were shown to have antimicrobial properties against various pathogens and were also found to help control bacterial infection, enhance immune response and treat digestive diseases^34^. The mechanisms involved in their antimicrobial effects remain largely unknown and appear to be strain specific^35,36^. The GRAS status of *L. casei* probiotics facilitated their use in healthcare and the food industry, making them good candidates to investigate antimicrobial activities against pathogens such as *Salmonella enterica*. In particular, the dairy strain BL23 of the species *Lacticasibacillus casei* (formerly referred to as *Lactobacillus casei*) is known for its beneficial anti-inflammatory properties^37,38^ and effects in host defense against pathogens^39,40^. However, to date no works on its antimicrobial activities against pathogens have been published.

Here, we aimed to evaluate the effects of the probiotic strain *L. casei* BL23 on the biofilm formation of *S.* Enteritidis. Our results showed that the cell-free supernatant (LC-CFS) and the cell lysate of *L. casei* BL23 have a strong antibiofilm activity against *S.* Enteritidis, whereas live cells have no significant effect. Furthermore, we demonstrated that membrane vesicles (LC-MVs) released by *L. casei* BL23 contribute to the antibiofilm activity of LC-CFS but have no impact on bacterial growth. In addition, we showed that LC-MVs have a strong antibiofilm effect at the early stage of biofilm formation and no effect on established biofilms of *S*. Enteritidis. Finally, we demonstrated that proteins associated with LC-MVs are responsible for the antibiofilm effects of the vesicles, and two peptidoglycan hydrolases (PGHs) were found to be involved in vesicle activities.

## Results

### The cell-free supernatant of *L. casei* BL23 contains lipid-based factors of high molecular weight, exhibiting antibiofilm activity against *S.* Enteritidis

**Figure 1:**
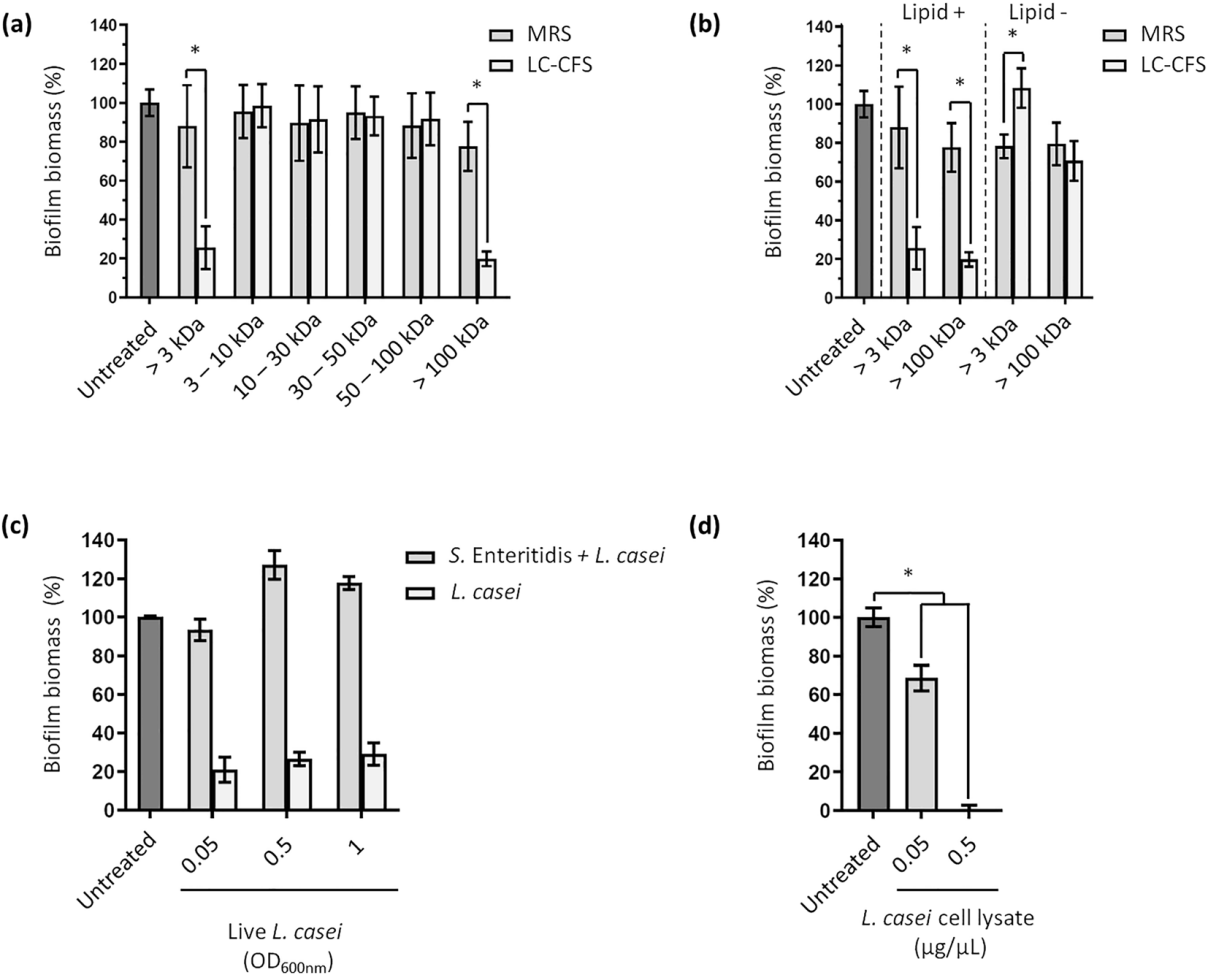
*L. casei* BL23 releases lipid components of high molecular weight in the supernatant with antibiofilm properties. (**a**) *S*. Enteritidis was grown in polystyrene microplates and treated with multiple fractions of *L. casei* cell-free supernatant (LC-CFS) and MRS medium (MRS). After 24 h of culture, the biofilms were quantified by crystal violet staining. The LC-CFS and MRS medium were fractionated by size-exclusion ultrafiltration, generating fractions with molecular weight ranging from 3 kDa to over 100 kDa. (**b**) The LC-CFS fractions exhibiting an antibiofilm activity and the corresponding MRS fractions were treated with a lipid adsorption matrix to selectively remove all the lipids. Biofilm biomasses of *S*. Enteritidis were then quantified by crystal violet staining after treatment with the fractions in the absence (Lipid −) and in the presence (Lipid +) of lipids. (**c**) *S*. Enteritidis biofilm formation was quantified after treatment with live *L. casei*. Please note that the control condition “*L. casei”* shows the biomass formed by *L. casei* BL23 in TSB without *S*. Enteritidis. (**d**) *S*. Enteritidis biofilm formation was quantified after treatment with lysed *L. casei*. All the results were normalized to the untreated conditions and expressed as a percentage.

To investigate the effect of *L. casei* BL23 on *S.* Enteritidis biofilm formation, we first quantified the biofilm biomass formed after treatment with several fractions of *L. casei* BL23 cell-free supernatant (LC-CFS). To this end, the LC-CFS and the growth medium of the bacteria (i.e. MRS) were fractionated by size-exclusion ultrafiltration to obtain several fractions ranging from 3 kDa to over 100 kDa. *S.* Enteritidis were then inoculated in 96-well plates and treated with the different fractions. Biofilms were finally stained by crystal violet and the relative biomasses were quantified by spectrometry at 595 nm (OD_595_). We observed a strong decrease in biofilm biomass formed by *S.* Enteritidis treated with the LC-CFS fractions containing only molecules larger than 100 kDa (hereinafter referred to as LC-CFS > 100) compared to the untreated fraction and the corresponding MRS control fraction (hereinafter referred to as MRS > 100) (Fig. 1a). Similarly, we observed that the LC-CFS fractions containing molecules over 3 kDa reduced the biofilm biomass formed by *S.* Enteritidis.

Next, we decided to remove all lipids from LC-CFS > 100 and LC-CFS > 3 fractions and their corresponding controls (MRS > 100 and MRS > 3) using a lipid removal reagent. The antibiofilm activity of the delipidated fractions (Lipid −) was then compared to the initial fractions (Lipid +) by crystal violet staining, as described above (Fig. 1b). Unlike with the initial fractions (Lipid +), the results showed that treatment with the delipidated fractions did not decrease the biofilm biomass formed by *S.* Enteritidis. In contrast, we saw a significant increase in biomass formation with the delipidated LC-CFS > 3 fraction compared to the control (MRS > 3) and the untreated fractions.

To further understand the mechanisms involved in the antibiofilm activity of the LC-CFS, we tested the effect of bacterial cells on the formation of *S.* Enteritidis biofilm. The biofilm biomasses were quantified after the treatment of *S.* Enteritidis with several concentrations of live (Fig. 1c) and lysed *L. casei* BL23 (Fig. 1d). We observed that the treatment with cell lysate resulted in a dose-dependent reduction of biofilm biomasses while treatment with live cells had no negative effects on *S*. Enteritidis biofilm formation (Fig. 1c,d). It is worth noting that the cell lysate did not significantly affect the bacterial biomass (OD_600nm_) of *S*. Enteritidis after 24 h of culture. Moreover, the “*L. casei”* control conditions (without *S.* Enteritidis) (Fig. 1c) showed that *L. casei* BL23 grown in TSB media formed little or no biofilm, and confirmed that live *L. casei* did not impact the biofilm formation of *S.* Enteritidis. We also noticed (Fig. 1d) that 0.5 µg/µl of cell lysate was sufficient to completely prevent the formation of *S.* Enteritidis biofilm. These data suggest that intracellular components with antibiofilm activities are released by *L. casei* BL23 in the supernatant.

Altogether, our results show that lipid components of high molecular weight with antibiofilm activities are released in the CFS by *L. casei* BL23. Previous work in *L. casei* BL23 has demonstrated that this bacterium releases membrane vesicles (MVs) in the supernatant^41,42^. MVs are lipid nanostructures of high molecular weight described in the literature to have a multitude of roles in the physiology of the bacteria. Importantly, MVs have been described to have positive or negative effects on the structure and on the development of the biofilm of several pathogenic bacteria^43–52^.

### Membrane vesicles released by *L. casei* BL23 inhibit the early stage of *S. enterica* Enteritidis biofilm formation without impacting bacterial growth

**Figure 2:**
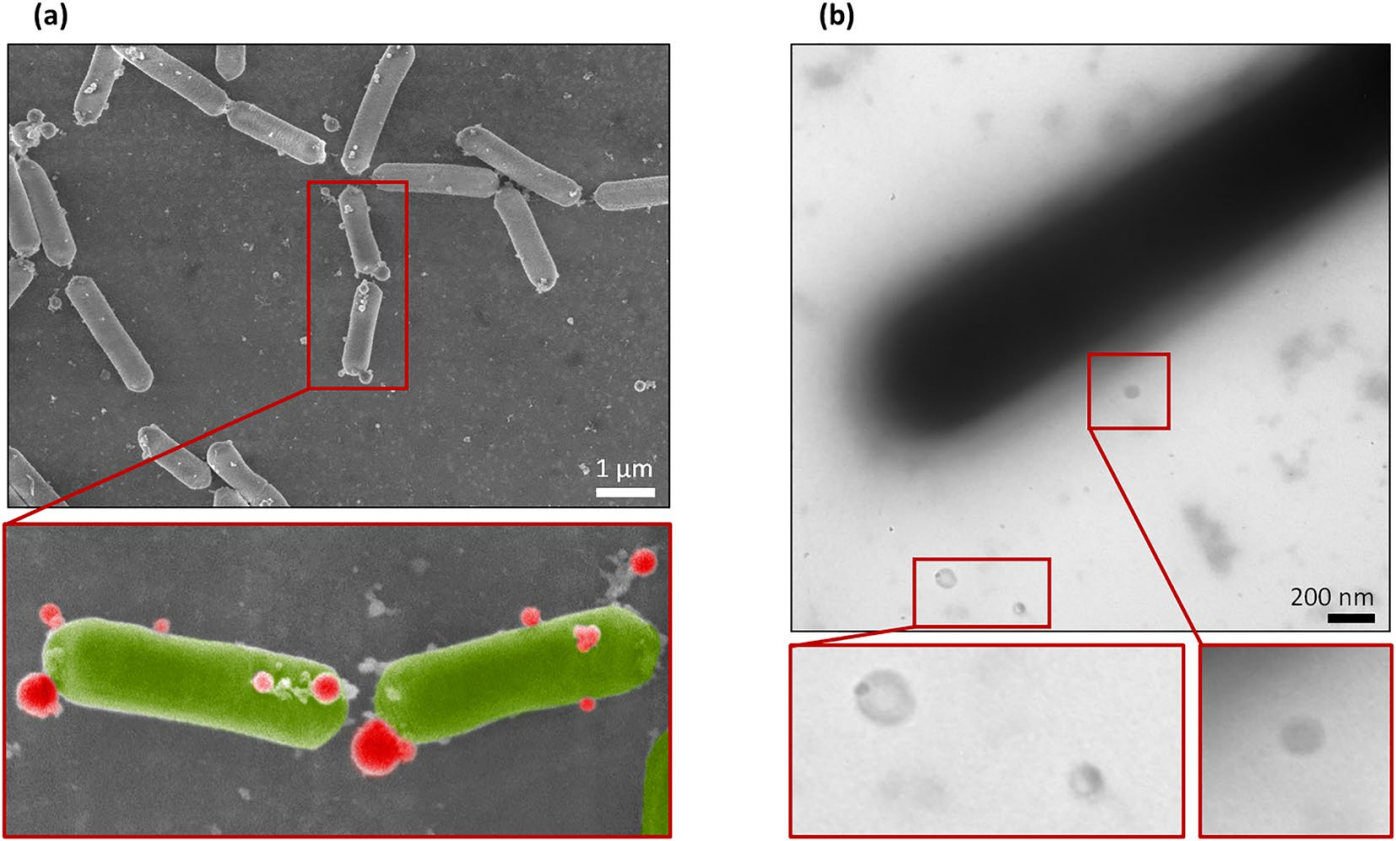
*L. casei* BL23 release membrane vesicles in the supernatant. SEM (**a**) and negative-staining TEM (**b**) images showing that MVs produced by *L. casei* BL23 are found on the surface of the bacteria and free in the supernatant. The bottom images show a magnified view of each EM images. MVs were colored in red and bacteria were colored in green in the SEM zoomed-in view for better identification.

**Figure 3:**
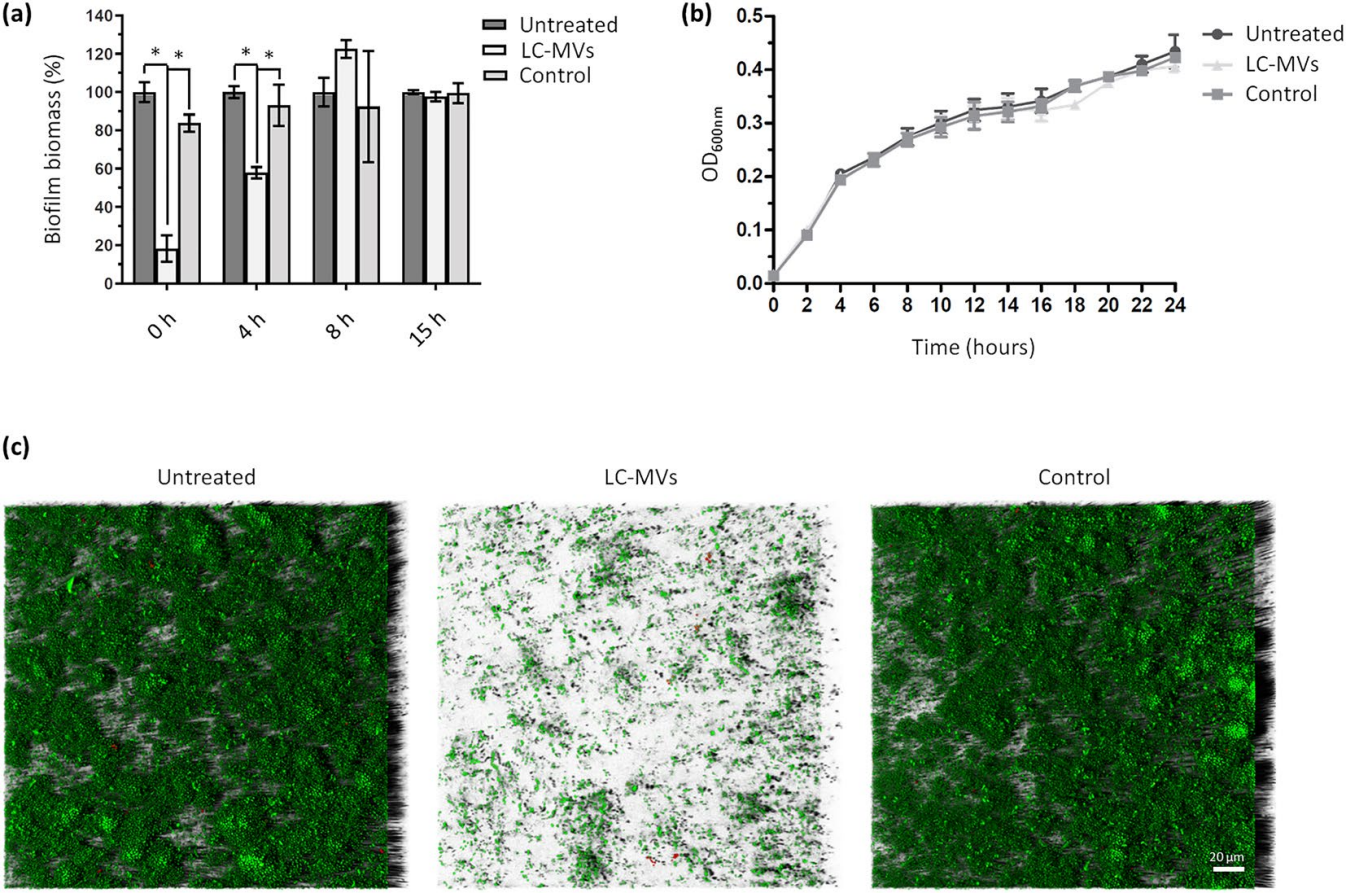
Membrane vesicles released by *L. casei* BL23 (LC-MVs) inhibit the early stages of biofilm formation without impacting the growth of *S*. Enteritidis. (**a**) *S*. Enteritidis was grown in polystyrene microplates and treated with LC-MVs (0.04 µg/µL) or a control fraction after 0, 4, 8 and 15 h of growth. After 24 h of culture, biofilms were quantified by crystal violet staining. The control corresponds to the fraction collected after carrying out the purification protocol on the culture medium alone (i.e., MRS medium). Results were normalized to the untreated conditions and expressed as a percentage. (**b**) Comparison of *S*. Enteritidis growth curves in the absence and in the presence of treatment with LC-MVs (0.04 µg/µL) and the control fraction. (**c**) The biofilms formed by *S*. Enteritidis after 24 h of culture in the absence or in the presence of treatment with LC-MVs (0.04 µg/µl) were stained (live/dead; SYTO 9/propidium iodide) and imaged by confocal laser scanning microscopy (CLSM). Green: total biomass. Red: dead cells.

The presence of MVs in the culture medium of *L. casei* BL23 was investigated using scanning electron microscopy (SEM) and transmission electron microscopy (TEM). Using SEM, spherical structures were observed in the medium and associated with the surface of bacteria after 24 h of culture (Fig. 2a). For easier identification and differentiation, these structures were colored in red and bacteria were colored in green in the zoomed-in view (Fig. 2a). MVs were also observed next to the bacteria by negative-staining TEM (Fig. 2b). In order to test whether the MVs released by *L. casei* BL23 (LC-MVs) contribute to the antibiofilm activity exhibited by CFS, MVs were purified and their effect on *S.* Enteritidis biofilm development was analyzed (Fig. 3a). For each condition, *S*. Enteritidis was treated with a final quantity of 0.04 µg/µL of MVs. When MVs were added to the culture of *S.* Enteritidis at the time of inoculation (0 h), we observed a reduction of 80% of the biofilm biomass compared to the untreated and control conditions after 24 h of growth. Moreover, the addition of MVs after 4 h of culture only induced a 40% decrease of the biofilm biomass compared to the control and untreated conditions. However, after 8 h and 15 h of culture, we noticed that MVs had no effect on the biofilm biomasses formed by *S.* Enteritidis. These results showed that *L. casei* BL23 MVs only affect the biofilm formation of *S.* Enteritidis when they are added early to the bacterial culture. This suggests that LC-MVs have no effect when the biofilm of *S.* Enteritidis is already established (Fig. 3a).

The effects of LC-MVs on the biofilm formation of several pathogenic and commensal bacteria was also investigated (Fig. S1). We observed that LC-MVs decrease the biofilm biomass of several bacteria with a variable degree of efficiency depending on the species. Furthermore, it was found that LC-MVs had an activity against both Gram positive and Gram negative bacteria (Fig. S1).

Interestingly, we noticed that LC-MVs had no effect on the growth curve of *S.* Enteritidis. This result proved that the antibiofilm properties of MVs were associated neither with bactericidal nor bacteriostatic activities (Fig. 3b). In addition, to assess the effect of MVs on the motility of *S*. Enteriditis a quantitative motility assay was performed. *S.* Enteriditis was mixed with or without LC-MVs and the diameter of the swim rings formed on 0.3% TSB agar plates was measured after 8 h of incubation. The results presented Fig. S2 suggest that the vesicles did not have a significant impact on the motility of *S*. Enteriditis.

The impact of MVs on the biofilm development of *S.* Enteritidis was further analyzed by confocal fluorescence microscopy. After 24 h of culture, we observed in the control and the untreated conditions that *S.* Enteritidis covered the bottom of wells with microcolonies attached to the polystyrene surface. In contrast, bacteria grown with LC-MVs were freely moving in medium and only a few were attached to the polystyrene surface (Fig. 3c).

Taken together, our results suggest that LC-MVs inhibit the early stage of *S.* Enteritidis biofilm formation. Furthermore, it is noteworthy that the antibiofilm properties of the MVs were associated with neither a bactericidal nor a bacteriostatic activity.

### The antibiofilm activity of *L. casei* BL23 membrane vesicles is sensitive to temperature and proteinase K digestion

**Figure 4:**
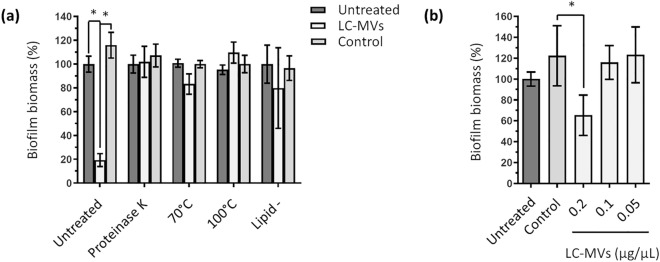
The antibiofilm activity of LC-MVs is sensitive to heat and proteinase K treatment. (**a**) LC-MVs and the control fraction were subjected to proteinase K treatment, heat inactivation (10 min at 70 °C and 100 °C) and lipid removal using a lipid adsorption reagent. Biofilm biomasses of *S*. Enteritidis were then quantified by crystal violet staining after adding the treated MVs and control fractions. (**b**) *S*. Enteritidis was incubated with increasing quantities of LC-MVs for 30 min at 37 °C before inoculation in polystyrene microplates. After 24 h of culture, the biofilm biomasses were quantified by crystal violet staining.

MVs consist of lipids, proteins and can also contain nucleic acids, sugars or other polymers. The composition of MVs depends on the organism that produces them and its physiological state^53–56^. To gain more information on the antibiofilm properties of LC-MVs, we aimed to identify the molecular nature of the factors responsible for their activity. To do this, we subjected the LC-MVs to heat treatment or proteinase K digestion before testing their influence on the biomass formed by *S.* Enteritidis biofilm. We observed that both treatments eliminated the antibiofilm activity of LC-MVs (Fig. 4a). Consistent with our previous results, we also found that the MV fraction lost its antibiofilm activity against *S.* Enteritidis biofilm after lipid removal. To examine whether lipids could be responsible for the antibiofilm effects, we tested the effect of liposomes on the biofilm formation of *S.* Enteritidis (Fig. S3). Since the main phospholipids in the cytoplasmic membranes of *L. casei* are phosphatidylglycerols (PG)^57^, PG-based liposomes were chosen for the experiment presented Fig. S3. Our results showed that the presence of liposomes increased the biofilm biomass of *S.* Enteritidis, suggesting that the lipid membranes of LC-MVs did not contribute to their antibiofilm activities (Fig. S3).

Next, we wanted to investigate whether a short pretreatment with LC-MVs would impact the biofilm formed by *S.* Enteritidis. To this end, *S.* Enteritidis was treated 30 min at 37 °C with increasing concentrations of LC-MVs. The bacteria were then washed twice to remove the LC-MVs before inoculation in 96-well plates. After 24 h of growth, the biofilm biomasses formed by *S.* Enteritidis were quantified by crystal violet staining. We observed a significant reduction in biofilm biomass for pretreatment with 0.2 µg/µL of LC-MVs (Fig. 4b). This result suggests that LC-MVs had an effect on *S.* Enteritidis cells which resulted in the inhibition of bacterial attachment to the polystyrene surfaces.

Altogether, these results suggest that proteins associated with LC-MVs are involved in antibiofilm activities against *S.* Enteritidis. In addition, it appears that LC-MVs are able to inhibit the attachment of *S.* Enteritidis to polystyrene surfaces, thus preventing biofilm development.

### The antibiofilm activity of *L. casei* MVs depends on two peptidoglycan hydrolases

The protein composition of LC-MVs was then analyzed in order to identify the proteins involved in their antibiofilm activities. LC-MVs were first compared to vesicles purified by density gradient ultracentrifugation (LC-pMVs) in order to control the quality of our samples. The quality of the LC-pMVs was also checked by negative-staining TEM (Fig. S4). The relative abundances of proteins present in the vesicles before (LC-MVs) and after (LC-pMVs) density gradient purification were analyzed and the results are shown Fig. S5a. The statistical analysis indicates that no proteins could be qualified as significantly differentially abundant, even with a false positive rate reaching 10% according to the Benjamini–Hochberg correction. These results showed that the protein composition of LC-MVs was not significantly different before and after density gradient purification. In agreement with these results, we observed that similar to LC-MVs, the vesicles purified by density gradient exhibited antibiofilm activity against *S.* Enteritidis (Fig. S5b). These results confirmed that LC-MVs and their protein content are involved in antibiofilm activities.

**Figure 5:**
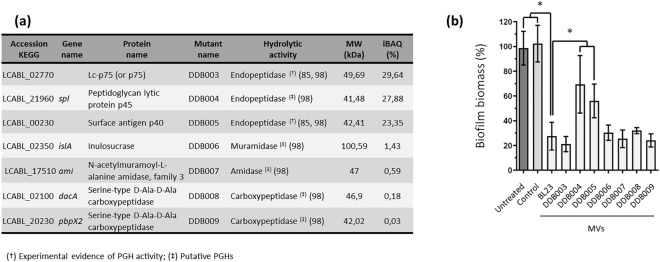
Mutation of two of the most abundant proteins in the vesicular fraction partially suppresses the antibiofilm activities of LC-MVs against *S*. Enteritidis. (**a**) List of the peptidoglycan hydrolases (PGHs) detected with the vesicles of *L. casei* BL23 and selected for mutagenesis. The column “Mutant name” indicates the names of the mutant strains obtained by the insertion of a non-replicative plasmid into genes of *L. casei* encoding PGHs. (**b**) The MVs of the mutant strains were purified and their antibiofilm activity against *S*. Enteritidis was analyzed, as described previously. For each condition, *S*. Enteritidis was treated with a final quantity of 0.04 µg/µL of MVs.

Based on the present analysis of the LC-MVs composition (Table S1), we selected and mutated genes that could be involved in the antibiofilm activity of the vesicles. Mutants were obtained by integration of a non-replicative plasmid in the bacterial genome. The selected genes encoding peptidoglycan hydrolases (PGHs) are listed in Fig. 5a and correspond to the most abundant proteins detected in the LC-MV fraction.

To investigate the PGH contribution to the antibiofilm activity of LC-MVs, we purified the MVs released by each mutant strain and quantified the effect of 0.04 µg/µL of the MVs on *S.* Enteritidis biofilm formation by crystal violet staining. The impact of the mutations on the protein profile of MVs was also analyzed by SDS-PAGE and Coomassie blue staining (Fig. S6). Interestingly, we saw that the biofilm biomass formed by *S.* Enteritidis treated with the MVs purified from DDB004 (LCABL_21960::pRV004) and DDB005 (LCABL_00230::pRV005) was twice as high compared to the biofilm biomass formed after treatment with parental strain MVs (referred to as BL23) (Fig. 5b). The MVs released by the strains DDB004 and DDB005 thus had a significantly lower antibiofilm effect on *S.* Enteritidis compared to the MVs released by the parental strain (Fig. 5b). Surprisingly, we observed that only the protein profile of the MVs produced by the strain DDB005 showed substantial differences compared to the vesicles of the parental strain BL23 (Fig. S6). The MVs of the strain DBB003 also showed noticeable differences in their protein profile; however these differences did not seem to have an impact on their activity. Furthermore, the results showed that the mutation of other genes encoding PGHs did not seem to impact the antibiofilm activity nor the protein profiles of LC-MVs (Fig. 5b and Fig. S6). Overall, these results showed that the PGHs LCABL_21960 and LCABL_00230 contribute to the antibiofilm activity of LC-MVs and suggest that their contribution to the antibiofilm activity involves different mechanisms.

## Discussion

Several bacteria of the *Lactobacillaceae* family have been shown to exert antimicrobial and antibiofilm activities against a broad spectrum of pathogenic and commensal bacteria^31,32^. The molecular mechanisms involved in the antagonistic activities of lactobacilli seem to depend on the species and remain largely unknown. The literature indicates that the biofilms of pathogens can be inhibited by live bacteria by competition, displacement or exclusion^31^. Moreover, cellular components and products secreted by lactobacilli have also been reported to exhibit strong antimicrobial and antibiofilm effects^32,58,59^. In this work, we showed that lipid components present in the high molecular weight fraction of LC-CFS displayed antibiofilm activities against *S.* Enteritidis. In addition, we observed that *S.* Enteritidis biofilm was not affected by live bacteria while cell lysate exerted a strong antibiofilm effect, suggesting that *L. casei* BL23 released cellular components with antibiofilm properties in the supernatant. Both these results led us to hypothesize that MVs released by *L. casei* BL23 could contribute to the antibiofilm effects of the CFS against *S.* Enteritidis.

Previous works have demonstrated that *L. casei* BL23 grown in standard conditions releases MVs in the supernatant^41,42^. MVs are spherical nanostructures bounded by a bilayered lipid membrane which contain various cellular components including proteins, nucleic acids and other polymers^53,54^. The composition of MVs depends on the species, the mechanism of biogenesis and the physiological state of the bacteria that produce them. This diversity explains that MVs can play various roles in metabolism, quorum sensing, immunomodulation and stress resistance (antibiotics, phage infection); they can also promote bacterial pathogenesis and horizontal gene transfer ^56,60^. Notably, it has been shown that MVs are a major component of the biofilm matrix and have a major impact on biofilm formation.

The presence and structural role of MVs in the biofilm matrix have been reported for many bacteria including *Pseudomonas aeruginosa*^43,61,62^, *Mycobacterium ulcerans*^63^, *Helicobacter pylori*^44,45^, *Myxococcus xanthus*^64,65^, *Streptococcus mutans*^46^. Moreover, the promoting effects of MVs on biofilm formation were proven for bacteria such as *Helicobacter pylori*^44^, *Pseudomonas putida*^48^, *Streptococcus mutans*^46,47^, *Aeromonas spp.*^49^, *Pseudomonas aeruginosa*^66^. In agreement with the literature, we found that the treatment of *S.* Enteritidis with liposomes increased the biofilm biomass after 24 h of culture. In contrast, the antibiofilm effects of MVs released by several bacteria have also been reported. The mechanisms of inhibition identified are diverse and seem to be strain dependent. In particular, it has been shown that MVs produced by *Paracoccus denitrificans* carry quorum sensing molecules which inhibit biofilm formation^67^. Similarly, the bactericidal effects of *Lactobacillus plantarum* and *Burkholderia thailandensis* MVs have been shown to inhibit biofilm development^50,51^. In *P. aeruginosa*, the secretion of a leucine aminopeptidase PaAP via MVs was also shown to disrupt biofilms^52^.

Our results showed that the addition of LC-MVs to a starting culture of *S.* Enteritidis, *H. alvei, C. freundii, S. aureus, B. subtilis, S. epidermidis, E. feacalis* reduces the biofilm biomass formed after 24 h of culture by the bacteria. For unknown reasons, we noticed that *S.* Enteritidis was the bacteria most affected by the antibiofilm activity of *L. casei* MVs with an 80% decrease in biofilm biomass. Furthermore, we observed that the earlier MVs were added to *S.* Enteritidis cultures, the lower the biofilm biomasses measured after 24 h of growth. This result suggests that LC-MVs do not disrupt already established *S.* Enteritidis biofilms. Interestingly, the antibiofilm properties of LC-MVs were not due to bactericidal or bacteriostatic activities against *S.* Enteritidis, suggesting a specific mechanism of inhibition. In addition, we showed that a short pretreatment of *S.* Enteritidis with LC-MVs was enough to reduce the biofilm biomass formed after 24 h of culture, suggesting that the vesicles interact with the bacterial cells. However, the nature of the interactions between LC-MVs and *S*. Enteritidis cells are yet to be characterized. Indeed, vesicles could attach to cell surfaces or fuse to the outer membrane of *S*. Enteritidis as previously described for other bacteria^68,69^. Taken together, these results suggest that LC-MVs impact the early stage of *S.* Enteritidis biofilm formation by inhibiting the initial attachment of the bacteria to polystyrene plate surfaces.

The antibiofilm activity of LC-MVs were inactivated by heat and Proteinase K digestion, suggesting that proteins associated with the vesicles were responsible for the MVs activities. This result led us to analyze the protein content of LC-MVs by MS-based proteomics to identify proteins that may contribute to the antibiofilm activity. The present MS-based proteomic analysis (Table S1) allowed us to identify the proteins associated with the LC-MV fraction. Strikingly, we noticed that peptidoglycan hydrolases (PGHs) were among the most abundant proteins of the LC-MV fraction. PGHs are enzymes responsible for the cleavage of covalent bonds within peptidoglycan chains and side-chains which is key for cell wall regulation during bacterial growth^70^. Among the proteins associated with LC-MVs, we identified the three main classes of PGH (amidases, glycosidases and peptidases)^70^. Recent studies have found that PGHs play a major role in MV biogenesis by triggering cell lysis^71^ or by creating holes in the PG which allow the extrusion of the cytoplasmic membrane^72–74^. Moreover, it was reported that some PGHs exhibit antibiofilm activities against pathogenic bacteria such as *S*. Typhimurium^75^ and Staphylococcals^76–83^. Lysozyme contained in egg albumen also displays a strong antibiofilm activity against *Gardnerella vaginalis*^84^. Thus, given the multiple roles played by PGHs, we sought to test the involvement in the LC-MV antibiofilm activity of the most abundant PGHs. To do this, the PGHs listed Fig. 5a were mutated by plasmid insertion. We observed that mutation of genes encoding the PGHs named LCABL_21960 and LCABL_00230 led to a significant reduction in antibiofilm activity of the MVs of both mutant strains compared to the MVs of the parental strain (referred to as BL23). These results showed that the PGHs LCABL_21960 and LCABL_00230 are directly or indirectly involved in the antibiofilm activity of LC-MVs. However, the mechanisms responsible for the suppression of the antibiofilm effect remain to be identified. PGHs could be involved in biofilm inhibition by directly interacting with *S.* Enteritidis^76–84^ and, as mentioned in the examples above, mutations of genes encoding the PGHs could lead to modifications in the composition of LC-MVs^72–74^. Indeed, we found that the protein profile of the MVs released by the strain DDB005 presented noticeable differences compared to the profile of the parental and DDB004 strains, suggesting that different mechanisms might be involved in the reduction of vesicle activity in these two strains.

The activity and localization of the protein p40 encoded by LCABL_00230 have been studied recently in *L. casei* BL23. Notably, the peptidoglycan hydrolase activity of p40 was proven experimentally^85^ and several other roles were attributed to the protein^85–87^. In particular, p40 was shown to bind to host cells and host macromolecules (mucin, collagen), activate cell receptors and induce signaling pathways^85–87^. Moreover, this enzyme was described to play a role in the growth and cell division of *L. casei* BL23*.* p40 was found on cell surfaces at the bacteria poles, and consistent with our results p40 has also been shown to be released into the environment freely^85^ or bound to extracellular vesicles^87^.

**Figure 6:**
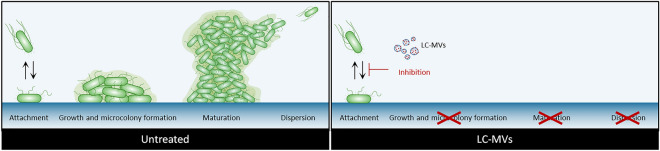
Schematic representation of the effect of LC-MVs treatment on *S.* Enteritidis biofilm formation. Proteins (in red) associated with LC-MVs inhibit the attachment of *S*. Enteritidis on polystyrene surfaces, preventing the formation of biofilm. Two peptidoglycan hydrolases (PGHs) were found to be involved in the antibiofilm activity of LC-MVs.

To conclude, we showed in this work that the MVs released by *L. casei* BL23 in the CFS exhibit a strong antibiofilm activity against *S.* Enteritidis without affecting cell growth. The antibiofilm activity of LC-MVs was inactivated by heat and Proteinase K digestion, suggesting the involvement of proteins. Furthermore, we showed that LC-MVs inhibits the early stage of *S.* Enteritidis biofilm formation. Finally, we found that the mutation of two peptidoglycan hydrolases resulted in a significant reduction of LC-MV antibiofilm activity (Fig. 6).

We believe that the antibiofilm activity of LC-MVs represents a promising therapeutic opportunity to fight negative biofilms in the medical sector and the food industry. The combination of LC-MVs to prevent biofilm development with more conventional treatments needs to be further investigated to improve the treatment of challenging negative biofilms.

## Methods

### Bacterial strains, plasmids and growth conditions

The bacterial strains and plasmids used in this study are listed in Table S2. Unless otherwise specified, *L. casei* strains were grown in Man-Rogosa-Sharpe medium (MRS; Conda) for 24 h at 37 °C under static conditions. *L. casei* mutant strains obtained by plasmid integration were selected in solid MRS (1.5% agar) and maintained in liquid MRS supplemented with 5 µg/mL of erythromycin. *Escherichia coli* DH5α was used as a cloning host and grown in LB medium at 37 °C with vigorous agitation (200 rpm). *E. coli* transformants were selected on solid LB (1.5% agar) supplemented with 100 µg/mL of ampicillin. *S.* Enteritidis were propagated in TSB (Tryptic Soy Broth) medium at 37 °C with shaking.

### Construction of plasmids and mutant strains

The construction of *L. casei* BL23 insertion mutants was adapted from Leloup et al. and Juan Rico et al.^88,89^. First, an internal fragment of each gene listed in Table S2 were amplified by PCR (Q5 High-Fidelity DNA polymerase NEB) with the corresponding primers using *L. casei* BL23 genome as a template (accession: NC_010999). The amplified fragments were then cloned into the suicide vector pRV300 previously digested with a combination of either EcoRI, HindIII or SacI restriction enzymes. The resulting plasmids (named pRV003, pRV004, pRV005, pRV006, pRV007, pRV008, pRV009) were then transformed in *L. casei* BL23 by electroporation using a GenePulser (BioRad), as described in Posno et al.^90^. Transformant bacteria were selected on MRS plates supplemented with 5 µg/mL of erythromycin. The correct integration of the plasmids was validated by PCR and sanger sequencing. The *L. casei* BL23 mutant strains obtained were named DDB003, DDB004, DDB005, DDB006, DDB007, DDB008, DDB009 accordingly.

### Biofilm quantification

*S.* Enteritidis was inoculated in TSB (Tryptic Soy Broth) medium at an optical density (OD_600nm_) of 0.05 measured at a wavelength of 600 nm. Bacteria were then grown in 96-well flat-bottom polystyrene plates (Greiner) at 37 °C under static conditions with or without treatment. After 24 h, *S.* Enteritidis biofilms were washed with distilled water (dH_2_O) and stained for 1 h at room temperature (RT) with a 0.5% aqueous crystal violet solution. Next, the stained wells were washed 3 times with dH_2_O and the crystal violet-stained biofilms were then solubilized in 95% ethanol solution. Finally, relative biofilm biomasses were quantified at OD_595nm_ using the Infinite 200 PRO microplate plate reader (Tecan). The biofilm biomasses of *S. marscescens, P. aerugunosa, H. alvei, C. freundii, S. aureus, B. subtilis, S. epidermidis* and *E. feacalis* were measured following the same staining procedure.

### Scanning electron microscopy (SEM)

Bacteria were fixed in 2.5% glutaraldehyde for 30 min and 10 µl of the fixed bacteria were then dropped onto a poly-L-lysine coated silicon wafer. After 30 min of drying, the bacteria were washed with water and post-fixed in 4% osmium tetroxide for 1 h. After gentle washing in distilled water, cells were dehydrated through a graded series of ethanol baths (from 30 to 100%) and dried by the critical point drying (CPD) method using a Leica CPD 030. Finally, the samples were coated with a thin carbon layer using a CRESSINGTON 308R and observed with a JEOL JSM 7600F scanning electron microscope (JEOL Ltd) at the DimaCell platform (http://www.dimacell.fr/).

### Transmission electron microscopy (TEM)

Negative staining was performed on the bacterial suspension and purified MVs (LC-pMVs) before observation by TEM at the DimaCell platform (http://www.dimacell.fr/). 10 µl of sample was dropped on collodion-coated and carbon-stabilized nickel microscope grids and left for 3 min to allow the bacteria or MVs to bind. Excess liquid was gently blotted with Whatman paper and stained with 10 µl of 1% (w/v) uranyl acetate solution for 10 s. The grid was dried and observed using a Hitachi H7500 transmission electron microscope (Hitachi Scientific Instruments Co., Tokyo, Japan) operating at 80 kV and equipped with an AMT camera driven by AMT software (AMT, Danvers, MA, USA).

### MV purification and cell free supernatant fractionation

For MV purification, we used a protocol adapted from a previous study^41^. A culture of *L. casei* (250 mL) was centrifuged at 4000 × g, 4 °C for 20 min. The supernatant was filtered through a 0.22 µm pore size filter (Nalgen Rapid-Flow) to remove remaining bacterial cells and concentrated by ultrafiltration at 4000 × *g*, 4 °C with 100 K Amicon^®^ Ultra-15 centrifugal filters (Merck Millipore). The concentrated supernatant was filtered through a 0.22-µm-pore-size filter before ultracentrifugation at 110,000 *g*, 4 °C for 2 h. The pellet of MVs was resuspended in sterile PBS (1X) and the protein concentration was quantified using a Bradford protein assay (Biorad) following the manufacturer’s instructions. Finally the MVs fraction was stored as aliquots at − 80 °C. This sample was named LC-MVs. As a control, the same procedure was applied to the MRS culture medium.

For further purification, an MV pellet was loaded on top of an iodoxinol gradient of 5–40% and subjected to ultracentrifugation at 100,000 × *g* for 18 h at 4 °C. The fraction containing the MVs was then collected and resuspended in PBS (1X) before a final ultracentrifugation at 110,000 × *g* for 2 h at 4 °C.Finally, the purified MVs were resuspended in sterile PBS (1X) and stored as aliquots at − 80 °C. This sample was named LC-pMVs.

The fractionation of the cell-free supernatant of *L. casei* BL23 (LC-CFS) began similarly to the purification of MVs described above. A culture of *L. casei* (250 mL) was centrifuged at 4000 × *g*, 4 °C for 20 min. The supernatant was filtered through a 0.22 µm pore size filter (Nalgen Rapid-Flow) to remove remaining bacterial cells and concentrated by ultrafiltration at 4000 × *g*, 4 °C with 3 K Amicon^®^ Ultra-15 centrifugal filters (Merck Millipore) to generate the LC-CFS. It is important to note that the resulting fraction contained all the molecules larger than 3 kDa; this fraction is also referred to as LC-CFS > 3 in the manuscript. Then the supernatant (LC-CFS > 3) was successively fractionated at 4000 × *g*, 4 °C using 100 k, 50 k, 30 k, 10 k Amicon^®^ Ultra-15 centrifugal filters. At each step the concentrate fractions were recovered and stored as aliquots at − 80 °C for later use.

### Cell lysate preparation

*L. casei* BL23 were washed twice with ice cold phosphate-buffered saline 1X (PBS). Cells were then harvested by centrifugation (5000 × *g*, 10 min), resuspended in ice cold PBS IX and transferred in sterile 2 ml-Precellys tubes pre-filled with inert 0.5 mm glass beads. Bacteria were then lysed with 2 cycles of 30 s using a Precellys24 homogenizer operating at 6500 rpm. The cell lysate was centrifuged (5000 × *g*, 10 min) and the supernatant was stored as aliquots at − 20 °C. Repeated freezing and thawing were avoided by using new aliquots in different experiments.

### Lipid removal procedure

Lipids and membrane vesicles were removed from samples using a lipid removal reagent (Cleanascite; Biotech Support Group) following the manufacturer’s instructions. Briefly, 1 volume of Cleanascite reagent was added to 4 volumes of sample; the solutions were then incubated at RT for 10 min with gentle shaking. After incubation, samples were centrifuged at 16,000 *g* for 1 min and supernatants were collected and stored as aliquots at − 80 °C.

### Protein digestion of *L. casei* BL23 MVs (LC-MVs)

*L. casei* BL23 MVs were subjected to protein digestion using proteinase K immobilized to agarose (P9290; Sigma-Aldrich). First, the purified MVs and the control fraction were incubated with 1 mg/mL of proteinase K-agarose at 37 °C for 1 h under agitation. The proteinase K-agarose was then removed by centrifugation (15 000 g, 1 min) and the digested fractions were finally stored at − 80 °C.

### Confocal laser scanning microscopy

*S.* Enteritidis was adjusted to an OD_600nm_ of 0.05 and cultured on 96-well black polystyrene plates with clear flat bottoms (Thermo Scientific) at 37 °C under static conditions. After 24 h, planktonic bacteria were removed and biofilms were washed twice with distilled water (dH_2_O). For visualization, biofilms were labeled with 5 μM of the cell-permeant nucleic acid stain SYTO-9 (Invitrogen) and with 20 μM of the cell-impermeant nucleic acid stain Propidium Iodide (Invitrogen) for 10 min in the dark at RT. Next, samples were observed at RT using a Leica confocal SP8 inverted microscope with a 63 × oil immersion objective lens (DimaCell platform; http://www.dimacell.fr/). A series of horizontal (xz) optical sections with a z-step of 0.75 μm were acquired for each biofilm. Samples were scanned at 3 randomly selected positions. 3D projections of each representative biofilm were reconstructed with the LAS X software (LEICA Microsystems, France).

### Mass spectrometry-based proteomic analyses

Three independent preparations of extracellular vesicles were analyzed. Proteins were solubilized in Laemmli buffer and heated for 10 min at 95 °C. They were then separated by SDS-PAGE (4–12% NuPAGE, Life Technologies), stained with Coomassie blue R-250 (Bio-Rad) before in-gel digestion using modified trypsin (Promega, sequencing grade) as previously described^91^. For each replicate, the two very intense bands were prepared separately from the rest of the sample. The resulting peptides were analyzed by online nanoliquid chromatography coupled to MS/MS (Ultimate 3000 RSLCnano and Orbitrap Exploris 480 for the second experiment, Thermo Fisher Scientific) using a 35-min gradient for intense bands and 120-min gradient for the rest of the samples. To this end, the peptides were sampled on a precolumn (300 μm × 5 mm PepMap C18, Thermo Scientific) and separated in a 75 μm × 250 mm C18 column (Reprosil-Pur 120 C18-AQ, 1.9 μm, Dr. Maisch). The MS and MS/MS data were acquired by Xcalibur (Thermo Fisher Scientific).

Peptides and proteins were identified by Mascot (version 2.7.0.1, Matrix Science) through concomitant searches against the Microscope database^92^ (*Lactobacillus casei* BL23 taxonomy, April 2021 download),the Uniprot database (*Saccharomyces cerevisiae* S288c and *Bos Taurus* taxonomies, June 2021 download), and a homemade database containing the sequences of classical contaminant proteins found in proteomic analyses (keratins, trypsin, etc.). Trypsin/P was chosen as the enzyme and two missed cleavages were allowed. Precursor and fragment mass error tolerances were set at respectively at 10 and 20 ppm. Peptide modifications allowed during the search were: Carbamidomethyl (C, fixed), Acetyl (Protein N-term, variable) and Oxidation (M, variable). The Proline software^93^ (version 2.1) was used for the compilation, grouping, and filtering of the results (conservation of rank 1 peptides, peptide length ≥ 6 amino acids, false discovery rate of peptide-spectrum-match identifications < 1%^94^, and a minimum of one specific peptide per identified protein group). Proline was then used to perform an MS1 label-free quantification of the identified protein groups based on razor and specific peptides. MS data have been deposited at the ProteomeXchange Consortium via the PRIDE partner repository^95^ with the dataset identifier PXD036342.

Statistical analysis was performed using the ProStaR software^96^ based on the quantitative data obtained with the three biological replicates analyzed per condition. Proteins identified in the contaminant, bovine and yeast databases, proteins identified by MS/MS in less than two replicates of one condition, and proteins detected in less than three replicates of one condition were discarded. After log2 transformation, abundance values were normalized using the variance stabilizing normalization (vsn) method, before missing value imputation using the slsa algorithm. Statistical testing was conducted with limma, whereby differentially expressed proteins were selected using a log2 (Fold Change) cut-off of 1 and a p-value cut-off of 0.01. The false discovery rate was then estimated using the Benjamini–Hochberg estimator.

Intensity-based absolute quantification (iBAQ, Ref.^97^) values were calculated from MS1 intensities of razor and specific peptides. The iBAQ values were normalized by the sum of iBAQ values in each sample, before summing the values of the three replicates to generate the final iBAQ value for each condition.

### Analysis of MVs by SDS-PAGE

The protein content of MVs was analyzed by one-dimensional sodium dodecyl sulfate (SDS)-polyacrylamide gel electrophoresis (PAGE). First, the protein concentration of the MV fractions was quantified using a Bradford protein assay (BioRad) following the manufacturer’s instructions. Then, 10 µg of MVs were solubilized in Laemmli buffer (BioRad) and heated for 5 min at 95 °C. The samples were loaded and separated on a 12% SDS-PAGE. Finally, gels were stained with Coomassie blue R-250.

### Quantitative motility assays

*S*. Enteritidis were grown overnight in TSB medium and 2 µl of the bacterial culture was mixed with 2 µL of PBS (Untreated) or 2 µL of LC-MVs (0,4 µg/µL) (LC-MVs) or 2 µL of a control fraction (Control). Then, 2 µl of each mix was spotted in the center of swim plates (0.3% agar, TSB medium) and plates were incubated facing upward at 37 °C. After 8 h of incubation, the swim ring diameters were measured.

### Statistical analysis

The results were calculated from a minimum of 3 biological replicates for all experiments. Statistical analyses were completed using GraphPad Prism (GraphPad Software). Unpaired t tests were used to compare the means of MRS and LC-CFS conditions in Fig. 1a,b, Fig. S1. A one-way ANOVA and Tukey’s multiple comparison test were used in Figs. 1c,d, 3a,b, 4b, Figs. S3, S5b. A two-way ANOVA and Tukey’s multiple comparison test were used in Fig. 2a. Error bars represent the standard deviation (SD) with * indicating that p-values are lower than 0.05. p-values lower than 0.05 were considered significant. For microscopy images, samples were scanned at 3 randomly selected positions and representative images were chosen.

## Supplementary Information

Supplementary Information.

Supplementary Table S1.

## Data Availability

Data supporting the findings of this study are available within the paper and its Supplementary Information files. All other data are available from the corresponding author on request.
