# Supplementary material for: Membrane vesicles released by Lacticaseibacillus casei BL23 inhibit the biofilm formation of Salmonella Enteritidis
Source: Sci Rep. 2023 Jan 20;13:1163. doi: 10.1038/s41598-023-27959-9 (PMC9859808; doi:10.1038/s41598-023-27959-9)
Supplement: Supplementary file 1 — Supplementary Information. [file 41598_2023_27959_MOESM1_ESM.pdf]

SUPPLEMENTARY INFORMATION FOR

Membrane vesicles released by *Lacticaseibacillus casei* BL23 inhibit the biofilm formation of *Salmonella* Enteritidis

David da Silva Barreira[a], Julie Laurent[a], Jessica Lourenço[a], Julia Novion Ducassou[b], Yohann Couté[b], Jean Guzzo[a] and Aurélie Rieu[a]

[a] *Université de Bourgogne Franche-Comté (UBFC), AgroSup Dijon, UMR PAM A 02.102, F-21000 Dijon, France.*

[b] *Univ. Grenoble Alpes, INSERM, CEA, UMR BioSanté U1292, CNRS, CEA, FR2048, 38000 Grenoble, France.*

Correspondence: Aurélie Rieu (aurelie.rieu@u-bourgogne.fr)

FIGURES

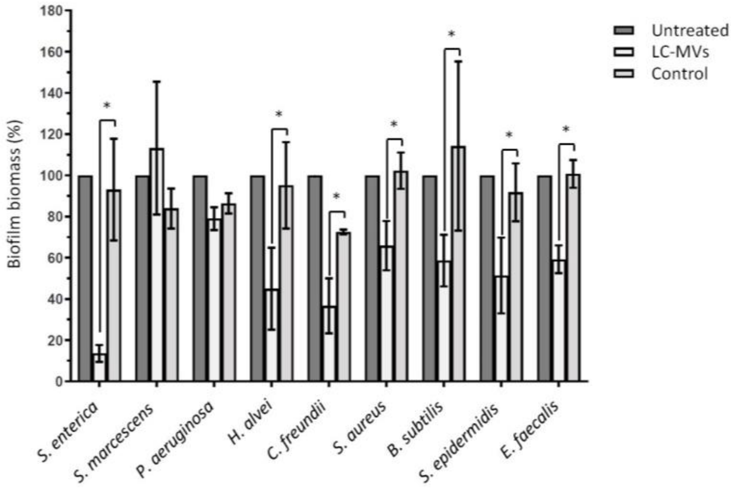

Fig. S1 MVs released by *L. casei* BL23 (LC-MVs) exhibit antibiofilm activities against a wide range of pathogenic bacteria. Bacteria were grown in polystyrene microplates and treated with LC-MVs (0.04 µg/µL) or a control fraction. The control corresponds to the fraction collected after carrying out the purification protocol on the culture medium alone (i.e. MRS medium). After 24 hours of culture, biofilms were quantified by crystal violet staining. Results were normalized to the untreated conditions and expressed as a percentage.

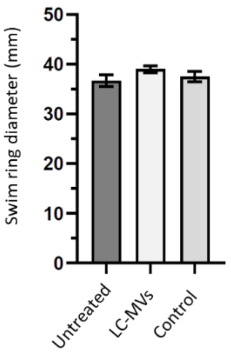

Fig. S2 LC-MVs does not significantly impact the cell motility of *S.* Enteritidis. 2 µL of an overnight culture of *S.* Enteritidis was mixed with 2 µL of PBS (Untreated) or 2 µL of LC-MVs (0,4 µg/µL) (LC-MVs) or 2 µL of a control fraction (Control). As mentioned above, the control corresponds to the fraction collected after performing the purification protocol on the culture medium alone (i.e. MRS medium). Next, 2 µL of the mix was spotted in the center of a swim plate (0.3% TSB agar) and the ring diameters were measured after 8 h of incubation at 37°C.

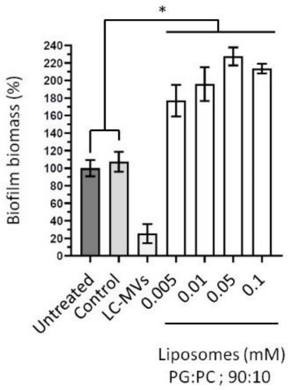

Fig. S3 Liposomes of similar lipid composition to LC-MVs promote *S.* Enteritidis biofilm development. *S.* Enteritidis was cultured for 24 hours with increasing concentration of Liposomes (PG/PC; 90:10 | Ref: CPG-501). Biofilm biomasses were then quantified by crystal violet staining.

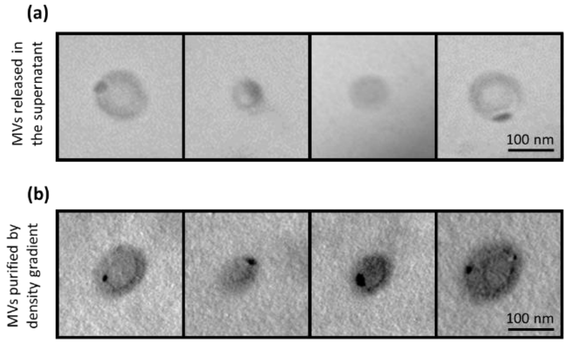

Fig. S4 Observation of MVs released by *L. casei* BL23 before and after density gradient purification. Negative-staining TEM images of MVs released in the supernatant by *L. casei* BL23 (a) and MVs collected after density gradient purification (b).

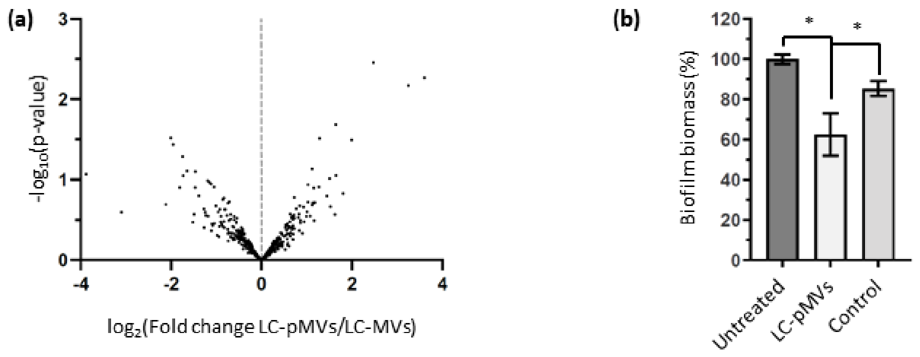

Fig. S5 No significant difference in protein abundance was obtained before and after purification of the LC-MVs by density gradient purification. (a) Volcano plot comparing the relative abundance of proteins identified in the vesicles obtained with the standard purification protocol (LC-MVs) to the proteins identified in the vesicles after density gradient purification (LC-pMVs). Red dots represent proteins with p-value < 0.01. Black dots correspond to non-significant change between conditions. (b) *S.* Enteritidis were cultured with vesicles (0.004 µg/µL) obtain after density gradient purification (LC-pMVs) or a control fraction (Control). After 24 hours of culture, biofilms were quantified by crystal violet staining as described previously.

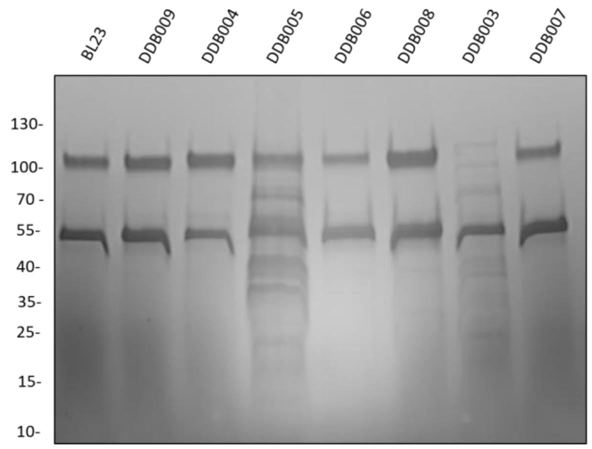

Fig. S6 SDS-PAGE protein profiles of MVs from parental and mutant *L. casei* BL23 strains stained with Coomassie blue. Molecular mass markers are indicated on the left (kDa).

TABLES

Table S1. MS-based label-free quantitative proteomic analysis of three independent preparations of *L. casei* BL23 MVs before (LC-MVs) and after (LC-pMVs) density gradient purification.

Table S2. Strains, plasmids and oligonucleotides used in this study.

**Strains**	**Relevant characteristic or description**	**Resistance(s)**	**Source or reference**
Escherichia coli			
DH5α	*F*– Φ80*lac*ZΔM15 Δ(*lac*ZYA_*arg*F) U169 *rec*A1 *end*A1 *hsd*R17(r_{k-}, m_k^+) *pho*A *sup*E44 *thi*_1 *gyr*A96 *rel*A1 λ–		[1]
Lacticaseibacillus casei			
BL23	Laboratory strains; sequenced genome		CECT 5275
DDB003	BL23 (LCABL_02770)::pRV003	Ery^r	This work
DDB004	BL23 *spl*(LCABL_21960)::pRV004	Ery^r	This work
DDB005	BL23 (LCABL_00230)::pRV005	Ery^r	This work
DDB006	BL23 *islA*(LCABL_02350)::pRV006	Ery^r	This work
DDB007	BL23 *ami*(LCABL_17510)::pRV007	Ery^r	This work
DDB008	BL23 *dacA*(LCABL_02100)::pRV008	Ery^r	This work
DDB009	BL23 *pbpX2*(LCABL_20230)::pRV009	Ery^r	This work
Salmonella enterica subsp. *enterica* Serovar Enteritidis			CIP 81.3
Serratia marscescens			CIP 58.14
Pseudomonas aeruginosa			CIP A22
Hafnia alvei			CIP 57.31
Citrobacter freundii			CIP 57.32
Staphylococcus aureus subsp. *aureus*			CIP 53.156
Bacillus subtilis subsp. *subtillis*			CIP 52.65
Staphylococcus epidermidis			CIP 53.124
Enterococcus feacalis			CIP 76.117

**Plasmids**	**Relevant characteristic or description**	**Resistance(s)**	**Source or reference**
pRV300	Insertional vector for lactobacilli	Amp^r, Ery^r	[2]
pRV003	pRV300 with a 692 bp fragment of the gene LCABL_02770 cloned at HindIII/ EcoRI sites	Amp^r, Ery^r	This work
pRV004	pRV300 with a 518 bp fragment of the gene LCABL_21960 cloned at HindIII/SacI sites	Amp^r, Ery^r	This work

pRV005	pRV300 with a 631 bp fragment of the gene LCABL_00230 cloned at HindIII/SacI sites	Amp^r, Ery^r	This work
pRV006	pRV300 with a 1070 bp fragment of the gene LCABL_02350 cloned at HindIII/SacI sites	Amp^r, Ery^r	This work
pRV007	pRV300 with a 517 bp fragment of the gene LCABL_17510 cloned at HindIII/EcoRI sites	Amp^r, Ery^r	This work
pRV008	pRV300 with a 637 bp fragment of the gene LCABL_02100 cloned at HindIII/EcoRI sites	Amp^r, Ery^r	This work
pRV009	pRV300 with a 444 bp fragment of the gene LCABL_20230 cloned at EcoRI site	Amp^r, Ery^r	This work

Ery^r, erythromycin resistant; Amp^r, ampicillin resistant; CECT, Colección Española de Cultivos Tipo; CIP, Collection de l'Institut Pasteur.

**Oligonucleotides**	**Sequence (5'_3')**
Construction of pRV003, pRV004, pRV005, pRV006, pRV007, pRV008, pRV009	
P003_FD	GGTATTAAGCTTCAGGATTCGTTGGTGCTGC
P003_RV	GGTATTGAATTCGAAGCGGCGGCATTTGAAGC
P004_FD	GGTACTGAGCTCCAACTGGCTCTTTGTCGCAAC
P004_RV	GATACGAAGCTTTTGGCTGGGGTTGGGGTTTC
P005_FD	GCACACAAGCTTCCAACAAAAGCGAAACCAATGC
P005_RV	CGTATTGAGCTCCGGCAACGGTCTTCAAAGC
P006_FD	CGTATTGAGCTCTGACGCCTTTACCCGAACATAC
P006_RV	GGAGTTAAGCTTGGCAAGGGCAACGAGTCTATG
P007_FD	GTTGATAAGCTTCGTGCGGTTAGGTCCTGGTTTG
P007_RV	CAGATTGAATTCGTCAGTTCGGTGCCTTTGTC
P008_FD	GGTATTAAGCTTCGCCAGCAATGTCGGTATCTG
P008_RV	GGTATTGAATTCTCGCATCACTGGTTCCCTTACG
P009_FD	GGTATTGAATTCCTTACGCAAACTAAAGCCGCTG
P009_RV	GGTATTGAATTCCGATCCGCCGTGAATACGAAG

FD, forward; RV, reverse

REFERENCES

1. Taylor, R. G., Walker, D. C. & McInnes, R. R. E. coli host strains significantly affect the quality of small scale plasmid DNA preparations used for sequencing. *Nucleic Acids Res.* **21**, 1677–1678 (1993).
2. Leloup, L., Ehrlich, S. D., Zagorec, M. & Morel-Deville, F. Single-crossover integration in the Lactobacillus sake chromosome and insertional inactivation of the ptsI and lacL genes. *Appl. Environ. Microbiol.* **63**, 2117–2123 , doi:10.1128/aem.63.6.2117-2123.1997 (1997).
