# Supplementary material for: Membrane vesicles released by Lacticaseibacillus casei BL23 inhibit the biofilm formation of Salmonella Enteritidis
Source: Sci Rep. 2023 Jan 20;13:1163. doi: 10.1038/s41598-023-27959-9 (PMC9859808; doi:10.1038/s41598-023-27959-9)
Supplement: Supplementary file 2 — Supplementary Table S1. [file 41598_2023_27959_MOESM2_ESM.pdf]

Table S1. MS-based label-free quantitative proteomic analysis of three independent preparations of *L. casei* BL23 MVs before (LC-MVs) and after (LC-pMVs) density gradient purification.

Legend	
(white)	Protein identified by MS/MS and quantified in the sample
(grey)	Protein identified and quantified in the sample by cross-assignment
(blue)	Imputed value - protein not identified in the sample

accession	protein name	gene name	coverage (%)	mol. weight (Da)	quantified peptides	log_2(Fold Change pMVs vs MVs)	limma p-value	log_2(abundance pMVs-R1)	log_2(abundance pMVs-R2)	log_2(abundance pMVs-R3)	log2(abundance MVs-R1)	log2(abundance MVs-R2)	log2(abundance MVs-R3)	iBAQ pMVs	iBAQ MVs
LCABL_02770	hypothetical protein		75,91	49693	21	-0,72	4,50E-01	34,851	32,068	35,483	34,597	34,277	35,701	9,28E+09	1,02E+10
LCABL_21960	peptidoglycan lytic protein P45	spl	97,28	41481	52	-0,33	6,36E-01	35,635	34,758	36,680	36,063	35,258	36,748	1,05E+10	9,63E+09
LCABL_00230	Surface antigen		66,42	42406	50	-0,14	8,48E-01	35,397	34,463	36,528	35,682	34,750	36,375	1,00E+10	8,07E+09
LCABL_24090	hypothetical protein		59,59	35368	26	-0,86	3,71E-01	30,715	29,194	32,304	32,085	30,243	32,473	7,47E+08	1,01E+09
LCABL_21780	hypothetical protein		88,64	38694	22	0,26	6,84E-01	32,597	31,186	31,752	30,881	32,141	31,746	1,20E+09	7,84E+08
LCABL_02350	Inulosucrase (Fragment)	islA	89,14	100594	113	0,26	6,77E-01	33,499	32,469	32,677	31,896	33,365	32,613	7,27E+08	4,95E+08
LCABL_30040	hypothetical protein		89,74	41342	44	-0,39	5,92E-01	31,327	29,618	31,780	31,214	30,757	31,912	4,55E+08	4,32E+08
LCABL_06650	hypothetical protein		80,53	31747	20	-0,46	5,22E-01	29,420	28,690	30,530	30,297	29,072	30,640	2,59E+08	2,60E+08
LCABL_21130	protein precursor (oligopeptide/dipeptide ABC transporter oligopeptide/dipeptide-binding protein)	oppA	80,76	61395	62	-0,25	6,79E-01	31,455	30,200	31,658	31,265	31,058	31,741	2,64E+08	2,29E+08
LCABL_17510	N-acetylmuramoyl-L-alanine amidase, family 3	ami	87,95	46996	38	-0,07	9,31E-01	30,626	29,544	31,895	31,221	29,354	31,712	2,50E+08	2,04E+08
LCABL_01640	hypothetical protein	yhgE	54,16	96914	39	0,09	8,83E-01	31,293	29,970	31,641	30,968	30,455	31,205	2,72E+08	1,87E+08
LCABL_03560	hypothetical protein		75	25796	9	-0,43	5,62E-01	28,904	27,116	29,471	28,938	28,414	29,422	1,52E+08	1,49E+08
LCABL_02460	transcriptional regulator	lytR	79,4	40173	38	-0,35	5,96E-01	30,441	28,718	30,395	30,440	29,704	30,460	1,49E+08	1,38E+08
LCABL_12590	Zn-dependent protease		81,94	30642	17	-0,39	5,40E-01	28,837	27,649	29,371	29,000	28,656	29,358	1,35E+08	1,30E+08
LCABL_18220	peptide ABC transporter substrate-binding	oppA	76,55	66461	61	-0,30	6,08E-01	30,704	29,409	30,692	30,481	30,303	30,932	1,26E+08	1,12E+08
LCABL_21830	hypothetical protein		75,35	30016	13	-0,15	8,12E-01	29,334	27,767	29,104	28,565	28,735	29,349	1,39E+08	1,11E+08
LCABL_27020	hypothetical protein		80,27	31171	19	-0,42	5,29E-01	28,066	27,313	29,107	28,557	28,008	29,176	9,91E+07	9,56E+07
LCABL_27170	hypothetical protein		72,81	50410	30	-0,21	7,79E-01	29,475	28,138	30,366	29,706	28,648	30,258	1,05E+08	8,94E+07
LCABL_02480	cell wall-associated hydrolase		79,39	23055	15	-1,11	4,29E-01	26,041	24,102	28,860	28,079	25,199	29,046	5,23E+07	7,58E+07
LCABL_22360	acyltransferase 3		49,37	26297	11	-0,51	4,62E-01	27,700	26,378	28,266	28,269	27,221	28,381	5,98E+07	6,31E+07
LCABL_02100	serine-type D-Ala-D-Ala carboxypeptidase	dacA	70,18	46903	44	-0,31	6,19E-01	29,562	28,253	29,637	29,556	28,955	29,856	6,86E+07	6,09E+07
LCABL_11320	Triosephosphate isomerase	tpiA	94,42	26980	25	0,07	8,94E-01	27,866	28,036	27,877	27,695	28,162	27,722	8,71E+07	5,59E+07
LCABL_22140	Wzr	wzr	67,11	32647	22	-0,08	8,99E-01	28,530	27,189	28,978	28,292	28,105	28,537	6,69E+07	5,30E+07
LCABL_11330	Enolase	eno	69,35	47111	41	-0,23	7,28E-01	28,371	27,892	29,257	29,352	27,858	28,986	5,89E+07	5,16E+07
LCABL_11310	Phosphoglycerate kinase	pgk	79,29	42220	34	-1,39	1,56E-01	26,435	26,962	29,188	29,144	27,612	29,990	2,71E+07	4,63E+07
LCABL_23150	phosphoglyceromutase	gpmA	75,98	25953	23	-0,01	9,90E-01	27,478	27,793	27,628	27,475	27,674	27,768	6,23E+07	3,89E+07
LCABL_10770	ATP-dependent Clp protease proteolytic	clpP	52,55	21525	14	-1,04	3,47E-01	26,226	23,037	27,093	27,178	25,408	26,892	2,42E+07	3,31E+07
LCABL_20460	hypothetical protein	yvhB	62,8	27330	14	-0,17	8,23E-01	28,080	25,730	27,848	27,470	26,704	28,000	4,26E+07	3,30E+07
LCABL_24540	50S ribosomal protein L7/L12	rplL	83,61	12518	12	-0,92	3,81E-01	24,883	24,443	26,988	27,494	24,360	27,233	1,93E+07	3,17E+07
LCABL_27800	hypothetical protein		69,8	31414	12	-1,26	3,93E-01	24,521	23,638	28,121	27,771	24,049	28,242	1,83E+07	3,09E+07
LCABL_24680	hypothetical protein		41,41	14002	8	-0,74	4,14E-01	25,415	24,475	26,927	26,948	24,832	27,260	2,33E+07	3,05E+07
LCABL_01530	hypothetical protein	yqcB	55,47	26019	11	-0,46	5,63E-01	27,154	25,397	27,828	27,426	26,335	28,002	3,00E+07	3,02E+07
LCABL_21740	xylanase/chitin deacetylase		65,06	36662	20	-0,10	8,59E-01	28,233	26,922	27,668	27,519	27,699	27,904	3,81E+07	2,89E+07
LCABL_15580	Elongation factor Tu	tuf	71,97	43573	39	0,04	9,41E-01	27,132	27,773	28,146	27,663	27,422	27,846	4,69E+07	2,84E+07
LCABL_01520	cell surface protein		73,54	27509	13	-0,20	7,53E-01	26,977	25,268	26,898	26,401	26,361	26,988	3,23E+07	2,69E+07
LCABL_15850	pyruvate kinase	pyk	74,15	62883	48	0,30	6,23E-01	28,219	28,149	28,976	28,837	27,329	28,278	4,35E+07	2,54E+07
LCABL_27630	tagatose 1,6-diphosphate aldolase	lacD2	78,61	36366	24	-0,43	4,83E-01	27,204	26,465	27,805	27,866	26,940	27,958	2,50E+07	2,44E+07
LCABL_05010	fructose-bisphosphate aldolase	fba	87,03	31502	25	-0,01	9,84E-01	26,700	27,223	27,276	27,187	26,897	27,147	3,91E+07	2,43E+07
LCABL_01070	phospholipase A2 family enzyme		68,24	19104	12	-0,28	6,03E-01	26,847	25,951	26,838	26,926	26,689	26,856	2,66E+07	2,35E+07
LCABL_26680	50S ribosomal protein L23	rplW	76	11560	14	0,25	6,78E-01	25,862	26,568	25,050	25,477	26,107	25,131	4,95E+07	2,30E+07
LCABL_11740	Spermidine/putrescine ABC transporter	potD	71,71	40696	29	-0,06	9,18E-01	28,174	26,725	27,368	27,232	27,568	27,645	3,13E+07	2,29E+07
LCABL_17030	bifunctional glycosyltransferase/transpeptidase	pbp1A	59,71	82791	46	-0,16	7,87E-01	28,574	26,940	28,168	27,822	28,193	28,157	2,63E+07	2,21E+07
LCABL_27160	L-lactate dehydrogenase (L-LDH)	ldh	81,29	35499	18	-1,08	3,72E-01	24,215	24,781	27,517	27,787	24,369	27,594	1,33E+07	2,18E+07
LCABL_11300	glyceraldehyde 3-phosphate dehydrogenase	gap-1	70,59	36707	15	-1,51	3,36E-01	24,066	22,970	27,961	27,572	23,816	28,154	1,17E+07	2,12E+07
LCABL_27110	DivIC protein (Fragment)	divIC	27,07	15075	4	-0,50	5,46E-01	25,339	23,732	26,286	25,702	24,592	26,577	1,88E+07	1,97E+07
LCABL_26650	50S ribosomal protein L22	rplV	71,79	12730	11	-0,14	7,89E-01	25,333	25,917	25,241	25,547	25,919	25,441	2,94E+07	1,94E+07
LCABL_17990	30S ribosomal protein S2	rpsB	75,95	29547	29	0,30	6,17E-01	27,132	27,542	26,124	26,426	27,139	26,328	4,20E+07	1,93E+07
LCABL_26540	30S ribosomal protein S5	rpsE	72,46	17535	12	0,00	9,98E-01	25,828	26,686	24,869	25,760	26,377	25,241	3,65E+07	1,92E+07
LCABL_26750	Elongation factor G	fusA	81	76861	58	0,23	7,30E-01	28,127	28,288	26,975	27,181	28,463	27,069	3,21E+07	1,84E+07
LCABL_13990	Outer membrane lipoprotein	plpB	37,09	30436	9	-1,73	8,88E-02	23,651	23,098	24,693	24,410	27,558	24,678	3,18E+06	1,79E+07
LCABL_00730	Tryptophan synthase beta chain	trpB	1,72	43821	1	-0,13	8,55E-01	27,011	26,060	27,904	27,235	26,273	27,850	2,09E+07	1,67E+07
LCABL_26760	30S ribosomal protein S7	rpsG	64,1	17924	17	-0,24	6,55E-01	25,318	26,235	25,975	25,886	26,078	26,276	2,43E+07	1,64E+07
LCABL_24550	50S ribosomal protein L10	rplJ	61,9	18177	18	-0,41	4,49E-01	25,242	25,784	25,750	26,455	25,647	25,893	1,90E+07	1,57E+07
LCABL_14260	peptide ABC transporter substrate-binding	oppA	56,96	60099	28	-0,51	4,28E-01	26,782	26,189	27,850	27,787	26,823	27,754	1,47E+07	1,54E+07
LCABL_26580	50S ribosomal protein L5	rplE	57,78	20168	12	0,14	7,98E-01	25,774	26,583	25,420	25,746	26,195	25,401	2,93E+07	1,50E+07
LCABL_08730	bifunctional glycosyltransferase/transpeptidase	pbp1B	38,01	37849	17	-0,24	6,82E-01	26,789	25,676	26,976	26,674	26,410	27,074	1,72E+07	1,47E+07
LCABL_26710	30S ribosomal protein S10	rpsJ	65,69	11736	8	0,17	7,43E-01	25,252	25,727	25,256	25,474	25,347	24,912	2,66E+07	1,47E+07
LCABL_15650	glutamine ABC transporter substrate-binding	glnPH2	29,69	52744	21	-0,49	4,50E-01	26,754	25,636	27,201	27,370	26,346	27,338	1,41E+07	1,46E+07
LCABL_26570	30S ribosomal protein S8	rpsH	55,3	14774	8	0,08	8,80E-01	25,248	26,180	25,156	25,256	25,811	25,265	2,64E+07	1,38E+07
LCABL_24520	PII-type proteinase precursor (lactocepin) (cell wall-associated serine proteinase) (LP151)	prtP	64,09	199982	82	-0,54	5,18E-01	28,582	26,709	29,382	29,069	27,761	29,475	1,27E+07	1,36E+07
LCABL_22950	3-oxoacyl-acyl-carrier protein] synthase II	fabF	77,34	42005	21	0,06	9,08E-01	25,951	26,061	26,697	26,469	25,634	26,416	1,99E+07	1,27E+07
LCABL_06730	hypothetical protein		64,81	24198	9	-0,52	5,09E-01	25,337	23,960	26,441	25,692	25,025	26,575	1,20E+07	1,24E+07
LCABL_26660	30S ribosomal protein S19	rpsS	56,99	10577	8	0,03	9,56E-01	24,426	24,999	24,422	24,823	24,649	24,290	2,09E+07	1,23E+07
LCABL_26690	50S ribosomal protein L4	rplD	56,52	22442	13	0,27	6,46E-01	25,870	26,633	25,342	25,663	26,160	25,210	2,60E+07	1,22E+07

LCABL_15540	30S ribosomal protein S15	rpsO	43,82	10300	4	0,19	7,49E-01	23,907	24,502	23,572	23,401	24,500	23,524	2,17E+07	1,22E+07
LCABL_07680	ABC transporter periplasmic protein		63,98	34121	18	-0,43	5,58E-01	26,040	24,912	26,832	26,798	25,346	26,926	1,21E+07	1,22E+07
LCABL_15600	Trigger factor	tig	77,08	49379	31	-0,37	4,77E-01	26,204	26,911	26,858	27,172	26,869	27,046	1,57E+07	1,22E+07
LCABL_12870	Glucose-6-phosphate isomerase	pgi	62,11	49323	39	0,15	7,83E-01	26,853	26,652	26,182	26,287	26,858	26,104	1,97E+07	1,21E+07
LCABL_24230	transcriptional regulator	lytR	68,75	39050	23	-0,25	6,79E-01	26,941	25,433	26,655	26,561	26,365	26,843	1,39E+07	1,18E+07
LCABL_30150	ArpJ protein	arpJ	22,2	52277	18	-0,41	5,43E-01	26,539	25,043	26,759	26,913	25,813	26,849	1,20E+07	1,18E+07
LCABL_20230	serine-type D-Ala-D-Ala carboxypeptidase	pbpX2	43,38	42020	16	0,16	7,77E-01	26,944	25,586	26,565	25,858	26,374	26,373	1,65E+07	1,10E+07
LCABL_28750	XalA precursor	xalA	63,07	111644	48	-0,12	8,46E-01	28,254	26,557	27,515	27,297	27,637	27,744	1,41E+07	1,09E+07
LCABL_26560	50S ribosomal protein L6	rplF	56,82	19302	12	0,30	6,54E-01	25,632	26,088	24,306	24,978	25,738	24,395	2,32E+07	1,07E+07
LCABL_26330	50S ribosomal protein L13	rplM	73,65	16549	10	0,22	6,97E-01	24,802	25,836	24,889	24,728	25,252	24,902	2,30E+07	1,07E+07
LCABL_14750	30S ribosomal protein S4	rpsD	70,44	23306	19	0,46	4,87E-01	25,563	26,430	24,999	24,806	26,096	24,711	2,39E+07	1,07E+07
LCABL_16010	histone-like DNA-binding protein HU	hbsU	73,63	9526	10	-0,49	6,07E-01	23,000	22,511	25,369	24,888	22,646	24,826	1,02E+07	1,06E+07
LCABL_07310	UDP-glucose 4-epimerase (galactowaldenase)	galE	91,84	36338	29	0,34	5,69E-01	26,146	26,627	25,486	25,418	26,476	25,331	2,11E+07	1,06E+07
LCABL_30540	glutamine-binding protein / glutamine	glnP	33,54	52877	13	-0,38	4,86E-01	26,477	25,473	26,440	26,517	26,420	26,596	1,11E+07	1,06E+07
LCABL_02700	alpha/beta hydrolase superfamily protein		67,67	33154	19	-0,05	9,28E-01	26,291	25,339	25,600	25,839	25,891	25,643	1,44E+07	1,05E+07
LCABL_11540	sugar ABC transporter periplasmic protein		59,5	48073	30	-0,42	5,60E-01	26,427	25,301	27,275	27,140	25,860	27,256	1,01E+07	1,01E+07
LCABL_30010	hypothetical protein		38,04	29248	11	-3,88	8,33E-02	19,432	18,195	26,226	26,147	22,790	26,545	3,80E+06	9,98E+06
LCABL_15990	30S ribosomal protein S1	rpsA	69,25	47649	24	-0,65	4,70E-01	24,815	25,283	27,237	27,008	24,964	27,325	9,03E+06	9,89E+06
LCABL_24610	50S ribosomal protein L1	rplA	49,78	24480	15	0,10	8,53E-01	26,066	26,497	25,603	25,625	26,346	25,894	1,77E+07	9,84E+06
LCABL_06680	hypothetical protein		67,12	22498	11	-0,46	4,76E-01	24,962	23,937	25,767	25,650	24,927	25,466	9,32E+06	9,68E+06
LCABL_26550	50S ribosomal protein L18	rplR	52,1	12969	6	0,20	7,64E-01	24,802	25,333	24,093	24,948	25,154	23,531	1,70E+07	9,65E+06
LCABL_10630	phosphoglucomutase	pgm	78,43	63500	48	0,21	7,52E-01	27,388	27,390	26,129	26,866	27,482	25,939	1,65E+07	9,56E+06
LCABL_27360	50S ribosomal protein L31 type B	rpmE2	59,04	9548	4	-0,21	6,77E-01	24,007	23,683	23,848	24,295	23,747	24,125	1,25E+07	9,54E+06
LCABL_11110	hypothetical protein		48,9	43721	17	-3,09	2,51E-01	26,979	16,160	26,623	25,927	26,398	26,710	8,19E+06	9,50E+06
LCABL_01370	D-lactate dehydrogenase	ldhD	66,07	36907	16	0,35	5,34E-01	26,216	25,812	25,054	25,227	25,728	25,071	1,85E+07	9,32E+06
LCABL_26440	50S ribosomal protein L17	rplQ	38,58	14229	5	0,16	7,87E-01	24,535	25,228	23,954	24,246	24,977	24,017	1,73E+07	9,03E+06
LCABL_24620	50S ribosomal protein L11	rplK	65,96	14820	12	-0,55	3,39E-01	24,232	24,389	24,861	25,700	24,500	24,940	8,83E+06	9,01E+06
LCABL_21770	hypothetical protein		40,94	19602	7	-0,17	7,89E-01	25,587	24,194	25,755	25,209	24,937	25,899	1,07E+07	8,66E+06
LCABL_28670	hypothetical protein	ykcF	11,98	72578	6	-0,32	6,11E-01	26,063	24,575	26,203	25,721	25,764	26,318	9,37E+06	8,64E+06
LCABL_30080	Periplasmic trypsin-like serine protease	htrA	48,19	44951	11	-1,81	1,24E-01	22,629	22,320	25,702	26,201	23,676	26,202	3,59E+06	8,64E+06
LCABL_25140	cysteine aminopeptidase C2 (Bleomycin	pepC2	69,87	50601	40	0,02	9,71E-01	26,481	26,552	26,039	26,349	26,543	26,125	1,38E+07	8,63E+06
LCABL_23200	Sortase	srtA	47,64	26180	15	-0,56	4,33E-01	24,535	23,771	25,559	25,681	24,212	25,650	7,80E+06	8,63E+06
LCABL_16370	inorganic pyrophosphatase	ppa	82,26	33789	24	-0,20	7,13E-01	25,107	25,911	25,672	26,009	25,421	25,845	1,31E+07	8,61E+06
LCABL_22930	(3R)-hydroxymyristoyl-ACP dehydratase	fabZ	25,34	15988	5	-0,19	6,97E-01	24,092	24,614	24,338	24,652	24,398	24,578	1,24E+07	8,44E+06
LCABL_26600	50S ribosomal protein L14	rplN	34,43	13003	5	0,04	9,48E-01	24,723	25,075	24,607	24,918	25,161	24,221	1,25E+07	8,23E+06
LCABL_28810	hypothetical protein		53,37	22606	10	-0,24	6,80E-01	24,958	23,672	24,764	24,523	24,420	25,181	9,69E+06	8,10E+06
LCABL_23710	hypothetical protein		32,62	15739	3	-0,47	5,29E-01	23,481	22,460	24,572	24,299	22,989	24,645	7,14E+06	7,36E+06
LCABL_10970	hypothetical protein		76,92	18773	9	-0,04	9,49E-01	24,185	24,491	25,187	24,674	23,816	25,494	1,05E+07	6,98E+06
LCABL_16930	ribonucleotide-diphosphate reductase subunit	nrdF	70,25	37827	14	0,39	5,38E-01	24,878	25,971	24,428	24,280	25,282	24,555	1,68E+07	6,73E+06
LCABL_04690	NADH peroxidase	npr	57,68	49830	18	0,24	6,30E-01	25,907	25,485	25,571	25,211	25,706	25,314	1,10E+07	6,62E+06
LCABL_24200	60 kDa chaperonin	groL	67,83	57428	24	-0,46	5,01E-01	25,444	25,425	26,466	26,950	25,127	26,640	6,57E+06	6,58E+06
LCABL_01680	phosphoketolase	xpk	69,36	89632	43	0,38	5,56E-01	26,687	27,257	25,845	25,729	27,073	25,837	1,36E+07	6,56E+06
LCABL_15700	1-phosphofructokinase	fruK	78,62	32121	19	0,17	7,57E-01	24,856	24,909	24,178	24,594	24,890	23,948	1,05E+07	6,18E+06
LCABL_18100	50S ribosomal protein L19	rplS	43,48	13377	6	0,70	3,79E-01	24,230	25,111	22,864	22,921	24,477	22,696	1,72E+07	6,11E+06
LCABL_06140	Mf4.1	mf4	48,46	32337	9	-0,06	9,14E-01	25,471	24,310	25,321	24,648	25,274	25,366	7,63E+06	6,03E+06
LCABL_22970	Malonyl CoA-acyl carrier protein transacylase	fabD	53,09	32018	11	0,03	9,66E-01	24,618	24,884	23,533	24,338	24,743	23,879	1,05E+07	5,99E+06
LCABL_12950	Prophage Lp2 protein 7		50	12309	5	-1,49	2,67E-01	21,248	20,069	24,136	24,325	20,985	24,616	2,80E+06	5,94E+06
LCABL_17980	Elongation factor Ts	tsf	62,8	31701	18	-0,29	5,74E-01	24,636	24,844	24,863	25,440	24,912	24,850	7,37E+06	5,92E+06
LCABL_18990	6-phosphogluconate dehydrogenase	gndA	73,09	52206	36	0,19	7,27E-01	26,608	26,234	25,845	25,754	26,477	25,897	9,80E+06	5,90E+06
LCABL_31160	hypothetical protein		34,46	134400	15	-0,39	6,67E-01	24,900	23,633	26,484	25,782	24,083	26,323	5,94E+06	5,79E+06
LCABL_26610	30S ribosomal protein S17	rpsQ	19,54	10184	2	0,13	7,97E-01	23,570	23,583	23,415	23,259	23,854	23,059	8,84E+06	5,75E+06
LCABL_26640	30S ribosomal protein S3	rpsC	37,73	24872	9	0,28	7,43E-01	24,179	25,737	23,197	23,709	25,299	23,276	1,33E+07	5,71E+06
LCABL_17090	hypothetical protein		29,01	18389	4	-0,17	7,80E-01	24,594	23,571	24,826	24,396	24,040	25,055	7,08E+06	5,66E+06
LCABL_26470	30S ribosomal protein S13	rpsM	49,59	13491	7	-0,29	6,51E-01	23,082	24,924	23,246	24,195	24,157	23,784	1,10E+07	5,64E+06
LCABL_04840	phage infection protein	pip	46,81	98735	28	-0,59	4,74E-01	25,852	24,630	27,052	26,814	25,282	27,212	4,95E+06	5,59E+06
LCABL_22210	RmlB	rmlB	73,61	38644	23	0,23	7,00E-01	25,626	25,920	24,843	25,014	25,919	24,771	9,99E+06	5,53E+06
LCABL_22460	peptide ABC transporter substrate-binding	oppA	45,93	59524	22	-0,38	5,80E-01	26,102	24,496	26,462	26,285	25,435	26,495	5,75E+06	5,49E+06
LCABL_28500	hypothetical protein	llpP	32,11	52993	8	-0,15	7,95E-01	25,544	24,175	25,197	24,773	25,274	25,321	6,60E+06	5,47E+06
LCABL_05450	cysteine synthase	cysK1	82,52	32718	18	-0,55	3,48E-01	24,136	24,083	24,703	25,532	24,188	24,866	5,18E+06	5,40E+06
LCABL_26700	50S ribosomal protein L3	rplC	60,48	22627	17	0,09	9,23E-01	24,863	25,383	22,294	23,691	25,171	23,411	1,16E+07	5,37E+06
LCABL_22960	3-oxoacyl-acyl-carrier protein] reductase	fabG	78,51	25277	16	0,35	5,86E-01	24,811	25,077	23,634	24,303	24,758	23,399	1,10E+07	5,29E+06
LCABL_27640	hypothetical protein	yqhA	69,97	32367	18	0,16	7,71E-01	25,048	25,455	24,536	24,816	25,283	24,462	9,57E+06	5,27E+06
LCABL_02140	oxidoreductase	ycgG	74,13	31618	16	0,28	6,52E-01	24,744	25,582	24,341	24,471	25,345	24,015	1,03E+07	5,20E+06
LCABL_22980	enoyl-acyl-carrier-protein reductase	fabK	69,82	34312	21	0,31	6,47E-01	24,865	25,673	24,046	24,544	25,308	23,815	1,08E+07	5,11E+06
LCABL_18750	glucokinase	glk	63,78	33904	13	-0,01	9,80E-01	24,480	24,981	24,172	24,567	24,847	24,260	8,21E+06	4,97E+06
LCABL_22200	RmlD	rmlD	74,73	32189	17	0,42	5,43E-01	25,192	25,730	24,021	24,341	25,393	23,961	1,09E+07	4,90E+06
LCABL_08780	glycerophosphoryl diester phosphodiesterase		37,44	49774	14	-0,03	9,52E-01	25,297	23,869	24,463	24,312	24,798	24,625	6,55E+06	4,84E+06
LCABL_18620	50S ribosomal protein L21	rplU	58,25	11318	5	0,10	8,53E-01	23,808	23,759	23,058	23,437	23,578	23,323	8,31E+06	4,72E+06
LCABL_00110	30S ribosomal protein S18	rpsR	43,59	9128	5	0,21	7,54E-01	22,487	23,888	22,317	22,262	23,499	22,304	1,00E+07	4,71E+06
LCABL_17780	Chaperone protein DnaK	dnaK	62,02	67564	35	-1,18	2,81E-01	24,839	22,895	26,723	25,851	24,856	27,281	3,00E+06	4,59E+06
LCABL_26460	30S ribosomal protein S11	rpsK	52,71	13772	7	0,14	8,12E-01	23,538	24,245	22,939	23,344	23,910	23,052	8,75E+06	4,48E+06
LCABL_16300	galactose mutarotase related enzyme		69,86	32630	15	0,27	6,95E-01	24,968	25,388	23,594	24,034	25,151	23,963	9,28E+06	4,43E+06
LCABL_20510	hypothetical protein		48,85	18567	6	-0,50	4,27E-01	23,201	22,896	24,299	24,103	23,323	24,479	4,33E+06	4,32E+06
LCABL_30300	oxidoreductase	iolS	75,73	34035	18	0,18	7,57E-01	24,596	25,500	24,497	24,258	25,245	24,556	7,89E+06	4,08E+06
LCABL_30430	extracellular solute-binding protein, family 1		51,06	46541	16	-0,45	4,72E-01	25,265	24,229	25,799	25,503	25,105	26,032	4,07E+06	4,04E+06

LCABL_26670	50S ribosomal protein L2	rplB	42,09	30305	12	-0,02	9,86E-01	24,464	25,572	22,546	24,137	25,237	23,257	8,37E+06	4,01E+06
LCABL_30530	glutamine-binding protein / glutamine	glnP	23,16	52772	13	-0,42	4,92E-01	25,284	24,016	25,237	25,016	24,975	25,798	4,16E+06	3,97E+06
LCABL_12210	hypothetical protein	camS	46,74	41596	13	-0,71	4,60E-01	24,459	22,473	25,645	25,333	23,619	25,744	3,27E+06	3,91E+06
LCABL_02860	beta-N-acetylglucosaminidase precursor	glcNAcase	53,6	61792	23	-0,24	7,18E-01	25,785	24,233	26,029	25,556	25,115	26,094	4,56E+06	3,90E+06
LCABL_22990	acyl-carrier protein	acpP	54,43	9014	4	-0,40	4,87E-01	22,003	22,551	22,645	23,094	22,101	23,194	4,92E+06	3,88E+06
LCABL_26500	Adenylate kinase	adk	61,93	23635	18	-0,10	8,63E-01	24,106	24,865	23,631	24,307	24,703	23,888	6,31E+06	3,79E+06
LCABL_13520	hypothetical protein	laaO	48,41	31130	11	-0,10	8,71E-01	24,695	23,187	24,779	24,178	23,952	24,841	4,77E+06	3,70E+06
LCABL_25920	substrate-binding protein	mtsC	62,62	35097	15	0,23	7,25E-01	24,946	23,993	23,735	23,236	24,863	23,891	5,42E+06	3,69E+06
LCABL_18150	30S ribosomal protein S16	rpsP	32,97	10489	3	-0,25	6,27E-01	22,216	22,480	22,547	22,905	22,376	22,698	4,85E+06	3,64E+06
LCABL_23020	(3R)-hydroxymyristoyl-ACP dehydratase	fabZ	44,14	15601	4	-0,41	4,26E-01	22,225	22,562	21,979	22,795	22,435	22,766	4,79E+06	3,63E+06
LCABL_26320	30S ribosomal protein S9	rpsI	45,38	14241	3	-0,13	8,20E-01	22,027	23,351	22,964	22,871	22,820	23,035	6,26E+06	3,61E+06
LCABL_21620	Prolinase	pepR	54,49	34167	14	0,73	4,11E-01	24,311	24,758	22,640	23,270	24,463	21,794	8,37E+06	3,56E+06
LCABL_06750	hypothetical protein		33,33	24315	4	-1,05	1,26E-01	22,919	21,546	21,073	22,848	22,635	23,198	2,97E+06	3,48E+06
LCABL_11620	phosphate acetyltransferase	pta	71,38	34930	14	-0,24	6,39E-01	24,345	24,413	24,688	24,951	24,493	24,709	4,44E+06	3,47E+06
LCABL_15840	6-phosphofructokinase	pfkA	61,76	34230	17	0,68	4,19E-01	24,819	25,458	23,288	23,631	25,092	22,816	8,35E+06	3,40E+06
LCABL_10410	ribosomal subunit interface protein	yfiA	40,54	20998	5	-0,26	6,14E-01	22,556	22,646	23,183	22,996	22,862	23,307	4,22E+06	3,37E+06
LCABL_05340	membrane alanine aminopeptidase	pepN	64,45	94496	37	0,02	9,72E-01	25,684	25,900	25,720	25,824	25,759	25,670	5,32E+06	3,33E+06
LCABL_13880	ATP synthase subunit beta	atpD	70,9	52926	22	-0,13	8,00E-01	24,173	24,656	24,612	24,530	24,773	24,517	4,38E+06	3,08E+06
LCABL_02540	type I signal peptidase-like protein	sipT	46,23	22543	8	-0,16	7,70E-01	24,257	23,320	24,286	24,120	23,816	24,410	3,83E+06	3,04E+06
LCABL_01350	D-alanine--D-alanine ligase	ddl	75,07	39020	20	-1,09	3,52E-01	20,778	23,510	25,331	24,905	23,118	24,870	3,10E+06	3,02E+06
LCABL_18980	Response regulator	rrp5	44,3	26318	8	0,29	6,74E-01	23,695	24,478	23,087	23,058	24,516	22,828	5,41E+06	2,98E+06
LCABL_30400	amino acid ABC transporter substrate-binding	glnP	33,27	58177	14	-0,61	3,91E-01	24,448	22,909	24,948	24,879	24,048	25,215	2,59E+06	2,90E+06
LCABL_21880	glutamine ABC transporter substrate-binding	glnH1	43,43	29714	10	-0,72	3,20E-01	23,245	22,197	24,257	24,333	23,194	24,317	2,36E+06	2,90E+06
LCABL_27730	L-2-hydroxyisocaproate dehydrogenase	hicD3	81,06	32571	15	0,21	7,27E-01	24,239	24,484	23,243	23,610	24,312	23,427	5,54E+06	2,88E+06
LCABL_26620	50S ribosomal protein L29	rpmC	41,18	7877	2	-1,27	2,29E-01	19,947	20,079	22,804	22,981	20,577	23,077	1,69E+06	2,88E+06
LCABL_27510	acetylglucosamine pyrophosphorylase (N-acetylglucosamine-1-phosphate uridyltransferase); glucosamine-1-phosphate N-	glmU	49,35	50059	13	0,28	6,97E-01	23,171	23,967	24,812	22,629	23,762	24,705	5,07E+06	2,86E+06
LCABL_17070	asparaginyl-tRNA synthetase	asnC	71,53	50061	20	0,38	6,70E-01	25,046	25,580	22,709	23,570	25,222	23,393	6,90E+06	2,83E+06
LCABL_01040	hypothetical protein		33,6	13707	3	-0,33	6,41E-01	22,497	21,474	23,408	23,302	21,876	23,196	2,96E+06	2,83E+06
LCABL_24770	hypothetical protein	cad	25,66	32733	6	-0,46	5,47E-01	23,145	23,035	24,867	24,359	23,093	24,974	2,84E+06	2,74E+06
LCABL_30340	EIIAB	manL	44,14	35318	12	0,11	8,24E-01	24,420	24,284	24,423	24,115	24,549	24,133	4,14E+06	2,72E+06
LCABL_12230	Glutamyl-tRNA(Gln) amidotransferase subunit	gatA	49,59	51337	17	0,35	6,24E-01	24,407	25,057	23,085	23,585	24,661	23,259	6,03E+06	2,65E+06
LCABL_22080	hypothetical protein		20,33	30479	3	-0,76	2,79E-01	21,167	21,436	22,553	22,944	21,468	23,020	2,28E+06	2,64E+06
LCABL_19150	50S ribosomal protein L20	rplT	22,03	13422	5	-0,59	5,63E-01	19,862	23,550	21,935	22,775	23,192	21,145	4,45E+06	2,61E+06
LCABL_15190	methylthioadenosine nucleosidase (nucleoside	pfs	64,41	24677	10	-0,27	5,99E-01	22,373	22,926	22,253	23,075	22,624	22,675	3,71E+06	2,55E+06
LCABL_24270	redox-sensing transcriptional repressor Rex	rex	53	24171	10	-0,25	6,21E-01	23,151	23,637	23,684	23,922	23,462	23,849	3,49E+06	2,55E+06
LCABL_21580	Dipeptidase	pepD3	50,96	52188	13	0,06	9,17E-01	24,000	24,731	23,942	24,013	24,575	23,917	4,38E+06	2,52E+06
LCABL_13300	hypothetical protein		58,38	38282	10	0,29	7,03E-01	24,519	23,053	25,383	24,228	23,249	24,623	4,18E+06	2,52E+06
LCABL_01760	hypothetical protein		30,95	23595	3	0,64	4,96E-01	22,892	22,250	25,780	23,250	22,475	23,268	6,28E+06	2,49E+06
LCABL_12650	extracellular protein		40,51	54175	9	0,11	8,39E-01	23,766	22,502	23,525	22,955	23,312	23,182	3,47E+06	2,43E+06
LCABL_11600	YcsE protein (Fragment)	ycsE	62,29	32151	16	-0,83	3,37E-01	22,913	23,888	20,923	23,495	24,043	22,671	3,14E+06	2,37E+06
LCABL_18780	Bifuntional dimerisation/transpeptidase	pbp2B2	49,44	77190	21	-0,26	6,45E-01	25,218	24,095	25,123	25,065	24,752	25,401	2,77E+06	2,35E+06
LCABL_27210	CBS domain containing protein		65,16	24889	11	0,30	6,50E-01	23,576	24,080	22,278	22,654	23,696	22,673	5,24E+06	2,35E+06
LCABL_00860	hypothetical protein		40,42	27166	4	-0,01	9,87E-01	23,734	22,655	23,793	23,096	23,314	23,800	3,16E+06	2,32E+06
LCABL_30110	hypothetical protein	yycH	31,37	53473	8	-0,12	8,28E-01	24,361	23,123	24,372	23,932	24,006	24,292	2,87E+06	2,31E+06
LCABL_08310	S-ribosylhomocysteine lyase	luxS	56,69	17267	5	0,22	7,27E-01	22,574	23,825	22,560	22,002	23,388	22,899	4,93E+06	2,30E+06
LCABL_11940	modification methylase LaaG	laaG	61,61	37043	12	0,93	2,21E-01	25,629	23,971	23,638	22,838	24,315	23,300	5,92E+06	2,27E+06
LCABL_20240	NH(3)-dependent NAD(+) synthetase	nadE	76	30073	15	0,24	7,13E-01	23,523	24,441	22,747	22,990	24,088	22,908	4,80E+06	2,26E+06
LCABL_08210	HAD-superfamily hydrolase, subfamily IA,		83,78	24639	14	0,18	7,57E-01	22,644	24,073	23,081	22,804	23,485	22,961	4,88E+06	2,25E+06
LCABL_26630	50S ribosomal protein L16	rplP	43,06	16037	8	0,04	9,75E-01	21,072	24,163	19,143	20,857	22,558	20,846	1,04E+07	2,23E+06
LCABL_24920	Glutamate--tRNA ligase	gltX	57,34	56853	28	0,01	9,81E-01	24,907	25,024	23,921	24,606	24,820	24,388	3,90E+06	2,22E+06
LCABL_26530	50S ribosomal protein L30	rpmD	27,87	6749	1	-0,15	8,24E-01	20,320	20,952	22,077	21,320	20,596	21,876	3,14E+06	2,20E+06
LCABL_31120	BacG protein	bacG	38,7	38447	12	0,01	9,84E-01	24,301	23,353	24,107	23,758	24,120	23,851	2,91E+06	2,18E+06
LCABL_11830	glucosamine--fructose-6-phosphate	glmS	65,84	65812	32	1,65	2,12E-02	25,729	26,332	25,966	24,596	25,025	23,470	9,16E+06	2,17E+06
LCABL_19420	protein maturation protease (peptidylprolyl	prtM	41,67	33498	7	-0,26	7,25E-01	23,576	23,004	24,601	24,244	22,778	24,946	2,42E+06	2,15E+06
LCABL_21470	peptide ABC transporter substrate-binding	oppA	23,38	59783	10	-0,45	4,87E-01	24,004	23,005	24,755	24,625	23,850	24,626	2,12E+06	2,15E+06
LCABL_16830	Formate--tetrahydrofolate ligase	fhs	50,45	58901	21	0,19	7,30E-01	24,572	24,749	24,143	24,608	24,658	23,619	3,61E+06	2,14E+06
LCABL_26520	50S ribosomal protein L15	rplO	38,36	15466	6	-0,26	6,36E-01	22,130	22,397	22,847	23,203	22,132	22,829	2,66E+06	2,11E+06
LCABL_09910	Major tail protein, phi13 family		39,53	23602	5	0,73	1,69E-01	23,598	23,821	23,274	22,739	22,817	22,952	1,98E+06	2,08E+06
LCABL_14990	bifunctional dimerisation/transpeptidase	pbp2B1	40	76853	22	-0,27	6,92E-01	24,751	23,425	25,465	25,106	24,194	25,156	2,25E+06	2,03E+06
LCABL_13530	hypothetical protein		19,68	25677	2	-1,75	5,02E-02	20,569	20,313	22,285	22,966	21,668	23,775	6,52E+05	1,97E+06
LCABL_29670	nicotinate phosphoribosyltransferase	pncB	47,7	52098	15	0,28	6,38E-01	23,913	24,017	22,980	23,048	24,097	22,915	3,49E+06	1,93E+06
LCABL_21220	hypothetical protein		51	127568	30	-0,82	3,94E-01	24,320	23,294	26,399	25,748	24,235	26,484	1,54E+06	1,92E+06
LACCB_2172	conserved protein of unknown function		14,29	5295	1	-0,27	7,28E-01	20,672	18,719	21,199	20,464	19,723	21,227	2,19E+06	1,90E+06
LCABL_13860	ATP synthase subunit alpha	atpA	40,08	55300	16	0,07	8,95E-01	23,873	24,863	23,850	24,138	24,377	23,852	3,64E+06	1,88E+06
LCABL_26030	outer membrane protein		21,77	64555	9	-0,68	5,16E-01	22,833	20,757	24,516	23,409	22,228	24,497	1,70E+06	1,84E+06
LCABL_17710	adenine phosphoribosyltransferase	apt	84,57	18897	12	-0,09	8,99E-01	22,781	23,782	21,582	22,817	23,325	22,268	3,64E+06	1,83E+06
LCABL_19780	hypothetical protein	ptsI	65,91	63409	31	1,16	1,26E-01	25,117	25,239	25,882	25,104	22,920	24,731	5,63E+06	1,83E+06
LCABL_10500	phosphate ABC transporter substrate-binding	pstS	19,93	30585	4	-0,32	5,83E-01	22,787	21,686	23,038	22,778	22,563	23,132	1,98E+06	1,81E+06
LCABL_27890	LemA precursor		34,65	22046	5	-0,52	4,95E-01	21,970	21,583	23,436	23,628	21,712	23,212	1,63E+06	1,80E+06
LCABL_26770	30S ribosomal protein S12	rpsL	34,78	15174	3	0,11	8,71E-01	21,628	23,455	21,712	22,224	22,448	21,802	4,46E+06	1,78E+06
LCABL_21540	GMP synthase glutamine-hydrolyzing]	guaA	65,76	57850	27	0,73	3,03E-01	25,088	25,604	23,697	23,970	24,763	23,462	5,29E+06	1,76E+06
LCABL_19480	arginyl-tRNA synthetase (arginine--tRNA ligase)	argS	53,57	62304	27	-0,94	5,13E-01	23,872	24,887	19,725	24,844	24,644	21,802	2,65E+06	1,76E+06
LCABL_19520	dihydroorotate dehydrogenase 1A	pyrDA	36,22	34040	8	0,36	6,03E-01	22,899	23,345	21,824	22,651	23,011	21,327	3,50E+06	1,72E+06

LCABL_07620	hypothetical protein		58,7	31858	14	0,12	8,87E-01	23,150	24,177	21,469	22,326	23,926	22,184	3,61E+06	1,71E+06
LCABL_16860	oxidoreductase		28,5	22767	4	-0,74	2,00E-01	21,328	21,828	21,950	22,802	21,839	22,683	1,67E+06	1,71E+06
LCABL_26140	phosphonate ABC transporter substrate-	phnD	53,02	34220	11	-0,52	5,31E-01	21,963	22,871	24,443	24,229	22,420	24,201	1,83E+06	1,71E+06
LCABL_08770	dipeptidase PepV	pepV	49,68	50313	14	0,02	9,65E-01	23,630	23,931	23,555	23,357	24,194	23,495	2,53E+06	1,69E+06
LCABL_08250	2,5-diketo-D-gluconate reductase	dkg	69,34	30879	13	0,31	7,83E-01	23,020	24,310	20,726	21,843	24,254	21,026	3,40E+06	1,68E+06
LCABL_12240	Aspartyl/glutamyl-tRNA(Asn/Gln)	gatB	60,71	53353	20	-0,44	6,47E-01	24,217	24,551	21,163	23,862	24,312	23,065	3,09E+06	1,68E+06
LCABL_08700	Ccpa protein (Catabolite regulator protein)	ccpA	71,47	36385	15	-0,19	7,51E-01	23,635	22,264	23,289	23,815	23,161	22,789	1,85E+06	1,67E+06
LCABL_09490	Leucine--tRNA ligase	leuS	67,62	91527	39	0,46	6,80E-01	24,355	25,982	22,024	22,655	25,193	23,144	5,24E+06	1,67E+06
LCABL_21120	(oligopeptide/dipeptide ABC transporter permease)	oppB	14,2	34748	1	-0,42	5,59E-01	22,358	20,990	22,645	22,239	21,616	23,397	1,69E+06	1,64E+06
LCABL_29790	exopolyphosphatase-related protein		62,58	33122	11	0,04	9,40E-01	23,263	23,504	23,031	23,063	23,329	23,294	2,77E+06	1,64E+06
LCABL_21820	glucose-1-phosphate thymidylyltransferase	rmlA	53,45	32341	13	0,16	7,81E-01	22,731	24,133	23,381	23,283	23,429	23,057	3,46E+06	1,63E+06
LCABL_20590	Serine--tRNA ligase	serS	65,81	48607	19	0,31	7,29E-01	24,112	24,584	21,985	22,427	24,615	22,706	3,25E+06	1,63E+06
LCABL_20490	NAD-dependent epimerase/dehydratase		52,83	23341	6	0,12	8,19E-01	22,101	23,081	22,448	22,537	22,486	22,244	3,15E+06	1,62E+06
LCABL_19170	translation initiation factor IF-3	infC	46,1	17303	7	0,64	3,35E-01	22,019	22,788	22,015	22,318	22,007	20,590	3,73E+06	1,62E+06
LCABL_18310	dihydroxyacetone kinase related enzyme		48,57	59631	17	0,34	5,94E-01	23,215	24,074	25,018	23,494	23,636	24,168	3,22E+06	1,61E+06
LCABL_22350	Wzd	wzd	15,86	34797	4	-0,35	5,97E-01	23,523	22,216	24,001	23,772	22,919	24,111	1,71E+06	1,60E+06
LCABL_12720	hypothetical protein		24,06	23272	3	-1,46	7,82E-02	20,252	20,073	21,860	22,323	21,144	23,098	8,18E+05	1,59E+06
LCABL_10450	cell-division associated ABC transporter ATP-	ftsE	63,6	25504	11	-0,22	6,51E-01	22,788	22,713	22,693	22,801	22,907	23,155	1,99E+06	1,49E+06
LCABL_08580	DltD	dltD	26,71	47810	9	-0,34	6,41E-01	23,710	22,193	24,317	23,856	22,973	24,414	1,60E+06	1,48E+06
LCABL_09920	phage protein		53,72	13710	3	0,11	8,29E-01	21,580	21,547	21,502	20,969	21,953	21,366	8,60E+05	1,48E+06
LCABL_17970	uridylate kinase	pyrH	53,56	25861	8	0,22	7,27E-01	22,759	23,336	22,035	22,578	23,179	21,709	2,71E+06	1,46E+06
LCABL_15730	acyltransferase 3		12,59	74290	7	-0,81	2,35E-01	23,182	22,141	23,413	23,718	22,897	24,565	1,16E+06	1,46E+06
LCABL_31070	(glucosamine-6-phosphate isomerase) (GNPDA) (GlcN6P deaminase)	nagB	59,07	26015	10	-0,10	8,51E-01	22,468	23,275	22,547	23,038	22,859	22,687	2,43E+06	1,45E+06
LCABL_21280	hypothetical protein	yjdB	25,23	23282	5	-0,36	6,21E-01	22,092	21,383	20,399	22,335	22,069	20,542	1,60E+06	1,45E+06
LCABL_11820	Phosphoglucosamine mutase	glmM	47,36	48795	12	0,04	9,42E-01	23,492	23,316	23,443	23,026	23,809	23,303	2,04E+06	1,45E+06
LCABL_13910	rod shape-determining protein MreB	mbl	50,15	34972	10	-0,05	9,44E-01	23,173	24,003	21,421	22,881	23,631	22,249	2,94E+06	1,44E+06
LCABL_20640	alpha-acetolactate decarboxylase	aldB	58,9	25938	10	0,17	7,67E-01	22,383	23,174	21,840	22,119	22,582	22,191	2,99E+06	1,39E+06
LCABL_01690	peptide ABC transporter substrate-binding	oppA	24,4	59672	12	-0,35	6,52E-01	23,799	22,607	24,829	24,563	22,961	24,776	1,40E+06	1,36E+06
LCABL_12470	hypothetical protein		58,91	23084	9	0,35	6,48E-01	22,940	23,279	21,192	21,759	23,179	21,409	2,82E+06	1,36E+06
LCABL_02760	Purine nucleoside phosphorylase deoD-type	deoD	49,16	25891	7	-0,47	4,55E-01	21,283	22,254	21,658	22,750	22,568	21,288	1,49E+06	1,36E+06
LCABL_06890	alpha, alpha-phosphotrehalase	treA	42,7	61934	19	0,33	6,25E-01	23,854	24,367	22,879	23,429	24,155	22,532	2,67E+06	1,35E+06
LCABL_25130	cysteine aminopeptidase	pepC1	71,3	49475	26	0,22	7,08E-01	23,294	24,475	23,255	23,153	23,849	23,366	3,00E+06	1,34E+06
LCABL_10960	hypothetical protein		54,47	27703	10	1,18	1,93E-01	23,150	24,365	21,490	21,329	22,923	21,199	6,21E+06	1,32E+06
LCABL_16100	hypothetical protein		46,43	30087	7	0,06	9,29E-01	21,739	22,976	21,760	22,648	22,608	21,037	2,29E+06	1,32E+06
LCABL_10180	thiamine biosynthesis lipoprotein	apbE	41,19	38239	9	-0,46	4,36E-01	23,329	22,214	23,368	23,361	23,094	23,824	1,33E+06	1,30E+06
LCABL_12370	hypothetical protein	ysaD	24,58	13304	2	-0,64	3,81E-01	21,353	20,492	22,499	21,894	21,440	22,920	1,15E+06	1,30E+06
LCABL_24340	hypothetical protein		5,23	19037	1	-0,44	4,03E-01	20,727	20,425	20,116	21,068	20,534	20,983	1,56E+06	1,29E+06
LCABL_12510	methionine aminopeptidase	pepM2	65,69	29956	13	0,17	8,22E-01	22,581	23,962	21,527	21,936	23,287	22,337	3,21E+06	1,28E+06
LCABL_08430	phosphoesterase, DHH family protein		65,09	34403	11	0,65	4,59E-01	22,920	25,406	22,192	22,710	23,373	22,477	6,73E+06	1,27E+06
LCABL_10210	hypothetical protein	ywcC	32,95	37646	7	0,07	9,07E-01	23,343	21,958	22,864	22,728	22,734	22,503	1,78E+06	1,27E+06
LCABL_27070	ATP-dependent zinc metalloendopeptidase	ftsH	30,77	77742	15	-0,43	5,26E-01	23,686	22,647	24,761	24,333	23,726	24,318	1,26E+06	1,26E+06
LCABL_11840	oligoendopeptidase F2	pepF2	59,9	67477	25	0,30	7,86E-01	24,166	24,799	21,485	23,978	24,281	21,290	2,81E+06	1,26E+06
LCABL_10620	thioredoxin reductase	trxB2	34,38	34497	9	-0,52	4,35E-01	22,138	21,289	23,132	23,140	22,092	22,893	9,15E+05	1,24E+06
LCABL_03070	peptide ABC transporter substrate-binding	oppA	23,22	60702	9	-0,89	3,34E-01	23,205	21,092	24,146	24,107	22,621	24,388	9,17E+05	1,23E+06
LCABL_23950	universal stress protein, UspA family	usp5	41,22	16220	4	0,03	9,61E-01	21,719	22,517	20,960	21,330	22,785	20,977	2,06E+06	1,23E+06
LCABL_02430	3-hydroxyisobutyrate dehydrogenase	mmsB	59,73	30727	14	1,27	1,26E-01	23,449	23,557	22,183	21,732	23,057	20,592	3,65E+06	1,23E+06
LCABL_13640	Dipeptidase D-type (U34 family)	pepD2	51,89	54315	20	0,43	6,07E-01	23,414	24,466	22,234	22,526	24,269	22,037	2,61E+06	1,22E+06
LCABL_16610	hypothetical protein		54,17	31984	11	0,10	9,18E-01	22,347	23,742	20,509	22,120	23,321	20,856	2,71E+06	1,22E+06
LCABL_02060	inosine-5-monophosphate dehydrogenase	guaB	59,8	52641	20	0,25	7,46E-01	22,853	24,190	22,065	22,806	23,741	21,812	2,65E+06	1,21E+06
LCABL_08230	alpha/beta hydrolase		24,84	34475	7	-0,26	6,67E-01	23,365	21,861	23,083	23,223	23,081	22,775	1,28E+06	1,18E+06
LCABL_14120	universal stress protein, UspA family	usp6	33,33	17986	6	-0,12	8,40E-01	21,584	22,669	21,359	21,884	22,336	21,742	2,04E+06	1,15E+06
LCABL_07900	alcohol dehydrogenase, zinc-binding domain		40	36211	7	-0,05	9,51E-01	22,063	22,952	20,453	21,415	22,962	21,239	2,23E+06	1,14E+06
LCABL_30060	small heat shock protein HSP19.5	hsp3	44,83	16520	5	-0,38	5,68E-01	21,515	21,655	22,788	22,820	21,381	22,908	1,25E+06	1,13E+06
LCABL_10560	YvlB	yvlB	46,65	53565	17	-0,24	6,55E-01	22,774	23,540	23,058	23,862	23,280	22,962	1,55E+06	1,11E+06
LCABL_27690	methionyl-tRNA synthetase	metS	46,97	74929	22	1,17	1,91E-01	25,127	23,334	26,539	23,314	23,788	24,382	3,41E+06	1,11E+06
LCABL_27460	Large-conductance mechanosensitive channel	mscL	21,95	13531	3	-0,60	2,90E-01	20,734	20,076	20,222	21,231	20,326	21,288	1,10E+06	1,08E+06
LCABL_27700	prolyl aminopeptidase	pepI	63,01	33389	11	0,61	4,73E-01	22,114	23,988	21,995	21,278	23,522	21,453	2,68E+06	1,07E+06
LCABL_15090	cell-division initiation protein (septum	divIVA	32,31	28461	5	0,15	8,25E-01	21,311	22,208	22,719	22,669	22,215	20,903	1,51E+06	1,06E+06
LCABL_03280	Low affinity penicillin-binding protein 5 (PBP5)	pbp5	23,98	71104	11	-0,42	4,74E-01	24,066	22,743	23,961	24,024	23,700	24,321	1,10E+06	1,05E+06
LCABL_22070	branched-chain amino acid aminotransferase	ilvE	49,56	37827	10	0,20	7,06E-01	23,407	22,630	22,930	22,975	22,806	22,599	1,69E+06	1,05E+06
LCABL_17890	transcription elongation factor NusA	nusA	56,35	45832	17	0,41	6,19E-01	22,101	23,916	22,095	21,759	23,713	21,396	2,13E+06	1,02E+06
LCABL_13810	Uracil phosphoribosyltransferase	upp	52,63	22809	8	1,17	3,22E-01	21,845	23,543	20,428	20,507	22,867	18,937	3,07E+06	1,01E+06
LCABL_27500	ribose-phosphate pyrophosphokinase	prs	33,02	35443	6	0,59	6,19E-01	22,784	23,814	20,013	21,553	23,349	19,943	2,61E+06	9,96E+05
LCABL_18290	Phosphate acyltransferase	plsX	41,06	36174	11	0,11	8,45E-01	22,670	23,002	22,140	22,197	23,043	22,248	1,68E+06	9,92E+05
LCABL_17210	hypothetical protein		48,48	29031	9	0,10	8,73E-01	21,082	21,997	21,365	21,219	22,241	20,691	1,50E+06	9,89E+05
LCABL_28660	ribose-5-phosphate isomerase A	rpiA	61,14	25052	9	-0,04	9,46E-01	22,408	22,861	21,985	22,065	22,907	22,392	1,58E+06	9,82E+05
LCABL_08400	glucose-6-phosphate 1-dehydrogenase	zwf	56,16	56008	19	-0,03	9,52E-01	22,971	23,658	22,618	23,274	23,322	22,750	1,69E+06	9,75E+05
LCABL_06160	Sortase SrtA	srtA	37,67	24942	5	-0,08	8,87E-01	22,062	21,755	22,816	22,509	21,702	22,666	1,32E+06	9,74E+05
LCABL_27530	pur operon repressor	purR	56,74	31173	13	-0,98	4,96E-01	22,625	23,268	17,681	22,363	23,113	21,030	1,76E+06	9,70E+05
LCABL_16470	YdgI	ydgI	41,23	23076	7	0,42	6,25E-01	21,420	22,715	20,798	21,134	22,635	19,919	1,84E+06	9,60E+05
LCABL_03610	hypothetical protein		52,27	14779	4	-0,22	6,84E-01	21,479	21,642	21,059	21,350	21,257	22,243	1,45E+06	9,45E+05
LCABL_15110	Isoleucine--tRNA ligase	ileS	44,07	105008	28	0,37	5,62E-01	24,578	24,716	23,252	23,558	24,400	23,492	2,06E+06	9,37E+05
LCABL_12560	UTP--glucose-1-phosphate uridylyltransferase	galU	52,96	33774	11	0,69	2,87E-01	22,976	23,330	22,593	22,488	23,034	21,317	2,01E+06	9,24E+05

LCABL_14810	valyl-tRNA synthetase	valS	47,61	100668	34	1,45	1,61E-01	25,595	25,393	23,063	22,465	24,944	22,287	3,07E+06	9,14E+05
LCABL_07090	alpha/beta hydrolase superfamily protein		18,53	28935	4	-0,61	3,16E-01	21,018	20,703	21,593	21,618	21,096	22,419	8,67E+05	8,99E+05
LCABL_19400	cell-cycle regulation Hit-like protein	hit	26,39	16179	2	0,05	9,25E-01	20,861	21,977	21,899	21,342	21,738	21,501	1,52E+06	8,78E+05
LCABL_15430	GTP-binding protein-BipA-EF-TU family	bipA	40,72	67911	20	1,08	2,79E-01	24,733	22,348	26,032	23,543	22,414	23,911	2,76E+06	8,76E+05
LCABL_06760	hypothetical protein		27,72	72660	13	0,70	3,08E-01	24,822	22,881	24,537	23,002	23,419	23,717	1,91E+06	8,67E+05
LCABL_18860	transcription elongation factor GreA	greA	37,18	17172	3	-1,18	1,03E-01	19,566	20,767	21,281	22,246	20,956	21,958	6,41E+05	8,61E+05
LCABL_17790	protein grpE (HSP-70 cofactor)	grpE	31,63	21776	4	-0,29	8,16E-01	20,414	20,178	22,272	22,598	18,283	22,860	6,41E+05	8,56E+05
LCABL_30950	Capsid protein	orf44	36,49	56530	14	0,13	7,92E-01	23,268	23,480	23,068	23,350	22,911	23,160	1,56E+06	8,50E+05
LCABL_18710	Aluminum resistance protein	Alu-2	20,1	44644	6	-1,23	2,76E-01	19,385	20,496	22,804	22,134	20,527	23,708	5,67E+05	8,49E+05
LCABL_02740	deoxyribose-phosphate aldolase	deoC	50,68	23014	7	0,02	9,78E-01	21,110	22,254	20,752	22,222	22,274	19,549	1,18E+06	8,41E+05
LCABL_27080	Hypoxanthine-guanine	hpt	54,14	20289	8	0,24	6,87E-01	21,417	22,787	21,882	21,363	22,159	21,853	1,87E+06	8,36E+05
LCABL_00830	hypothetical protein		23,48	41635	6	-1,15	1,07E-01	20,761	21,716	22,249	22,581	22,039	23,561	6,34E+05	8,28E+05
LCABL_27400	DNA-directed RNA polymerase subunit delta	rpoE	18,83	25162	4	0,04	9,66E-01	19,241	20,704	19,195	20,003	21,075	17,949	1,10E+06	8,21E+05
LCABL_02870	mannose-6-phosphate isomerase	manA	44,58	36016	8	0,01	9,89E-01	22,423	22,503	21,473	21,903	22,690	21,784	1,30E+06	8,19E+05
LCABL_01860	phosphoglycerate dehydrogenase	serA2	48,75	33599	9	0,99	2,87E-01	21,899	22,954	21,664	21,224	22,870	19,438	1,72E+06	8,17E+05
LCABL_03350	hypothetical protein	ypaG	10,85	14879	1	-0,45	5,74E-01	19,691	20,977	22,736	21,478	21,430	21,857	1,32E+06	8,14E+05
LCABL_01770	methanol dehydrogenase-like protein		13,96	47712	5	-0,13	8,46E-01	22,342	21,814	23,035	22,053	21,895	23,637	1,03E+06	8,13E+05
LCABL_17080	aspartate aminotransferase	aspB	42,2	42486	7	-0,63	5,63E-01	20,931	20,546	21,981	23,224	19,248	22,861	3,84E+05	8,13E+05
LCABL_07140	iron-dependent peroxidase		65,18	35147	12	0,12	8,75E-01	22,341	23,225	20,799	21,489	22,948	21,562	1,71E+06	8,10E+05
LCABL_18680	glutamine synthetase	glnA	47,31	50396	12	-0,01	9,89E-01	23,188	23,345	21,434	22,405	23,279	22,311	1,48E+06	8,05E+05
LCABL_21810	RmlC	rmlC	48,42	21456	7	-0,25	7,96E-01	19,684	21,760	22,151	22,367	19,669	22,309	1,19E+06	8,02E+05
LCABL_08550	D-alanine--poly(phosphoribitol) ligase subunit 1	dltA	33	56218	11	0,33	5,98E-01	22,363	23,157	22,082	22,513	22,778	21,314	1,57E+06	7,93E+05
LCABL_23960	Repressor (Gp132 protein)	Cpg	10,85	13986	1	-1,95	3,55E-02	19,339	18,526	19,400	21,586	19,367	22,156	2,12E+05	7,85E+05
LCABL_02040	GTP-dependent nucleic acid-binding protein		39,67	40293	8	-0,64	5,32E-01	20,214	21,733	23,662	23,059	21,034	23,434	8,41E+05	7,69E+05
LCABL_22940	acetyl-CoA carboxylase, biotin carboxyl carrier	accB	57,72	15935	3	-0,32	6,43E-01	20,534	21,155	19,289	21,029	21,029	19,871	1,16E+06	7,68E+05
LCABL_31000	ribose-phosphate pyrophosphokinase	prsA	45,75	33665	9	0,33	6,47E-01	21,892	22,887	21,150	21,343	22,694	20,903	1,52E+06	7,49E+05
LCABL_10070	MalY	malY	30,17	44728	9	-0,13	8,78E-01	21,444	22,478	24,096	23,084	21,522	23,815	1,08E+06	7,44E+05
LCABL_12770	Xanthine phosphoribosyltransferase	xpt	33,33	21353	6	-0,62	5,29E-01	18,869	22,451	19,230	20,898	21,276	20,230	1,99E+06	7,37E+05
LCABL_25160	hypothetical protein		5,03	44227	2	-0,92	2,34E-01	20,873	19,528	21,462	22,372	20,569	21,671	4,83E+05	7,36E+05
LCABL_25040	Internalin-J precursor	inlJ	21,75	44767	5	-1,24	2,51E-01	21,411	18,395	22,506	22,573	21,296	22,163	4,70E+05	7,31E+05
LCABL_24350	L-asparaginase	asnA1	18,52	34987	4	0,06	9,22E-01	20,545	22,282	21,334	20,834	22,018	21,117	1,38E+06	7,26E+05
LCABL_22920	Biotin carboxylase	accC	59,91	49023	17	0,41	6,72E-01	23,459	23,627	20,714	22,105	23,393	21,078	1,69E+06	7,26E+05
LCABL_27770	hypothetical protein		40,91	34911	11	0,15	8,34E-01	21,622	22,687	20,788	21,546	22,293	20,821	1,48E+06	7,19E+05
LCABL_13380	hypothetical protein		38,74	12023	2	-0,04	9,50E-01	21,193	19,741	22,068	21,096	20,605	21,435	9,42E+05	7,16E+05
LCABL_01100	50S ribosomal protein L9	rplI	41,06	16962	5	0,39	5,57E-01	21,340	20,994	21,566	21,769	21,181	19,792	1,02E+06	6,97E+05
LCABL_05050	glutamine amidotransferase class-I:peptidase		29,6	27762	4	0,12	8,32E-01	21,669	21,466	20,711	21,309	20,700	21,485	1,36E+06	6,95E+05
LCABL_05330	cell envelope-associated proteinase PrtR	prtR	24,27	192861	26	0,24	6,72E-01	24,878	23,588	24,300	23,986	24,402	23,645	1,02E+06	6,80E+05
LCABL_02890	tagatose-6-phosphate ketose/aldose isomerase	agaS	47,07	42754	10	-2,11	2,01E-01	16,620	21,409	23,304	22,613	21,386	23,680	5,48E+05	6,70E+05
LCABL_29080	hypothetical protein	STY3947	33,33	30288	6	-0,36	6,78E-01	21,263	22,463	19,467	20,533	22,255	21,484	1,22E+06	6,53E+05
LCABL_26820	DNA-directed RNA polymerase subunit beta	rpoB	53,54	133682	39	0,62	6,73E-01	24,249	25,277	20,230	22,059	24,881	20,957	1,74E+06	6,39E+05
LCABL_08910	peptidyl-prolyl cis-trans isomerase	ppiB	18,56	21296	2	0,45	4,65E-01	20,829	21,304	20,194	20,568	20,866	19,542	1,36E+06	6,33E+05
LCABL_01490	hypothetical protein		20	73957	9	0,57	4,72E-01	24,152	22,610	22,771	23,000	23,565	21,273	9,78E+05	6,17E+05
LCABL_15010	UDP-N-acetylmuramoylalanine--D-glutamate	murD	37,47	50444	12	0,30	5,77E-01	22,286	23,139	22,569	22,173	22,642	22,291	1,29E+06	6,15E+05
LCABL_18880	hypothetical protein	ygcC	16,45	42156	5	-0,89	2,83E-01	21,544	20,259	22,360	23,090	20,991	22,751	4,10E+05	6,14E+05
LCABL_08860	N-acetylglucosamine catabolic protein	nagD	42,64	28404	8	0,20	7,50E-01	21,189	22,116	20,814	21,369	21,694	20,466	1,20E+06	6,05E+05
LCABL_26070	cell surface protein		7,65	81078	5	-0,27	6,64E-01	22,374	20,910	22,521	22,065	22,119	22,424	6,65E+05	6,01E+05
LCABL_17380	Putative phosphotransferase LCABL_17380		47,83	30900	9	0,27	7,64E-01	20,991	22,783	20,173	20,426	22,531	20,174	1,28E+06	6,00E+05
LCABL_14850	rod shape-determining protein MreB	mreB	41,14	34983	9	0,14	8,11E-01	21,795	22,591	21,403	21,802	22,199	21,379	1,16E+06	5,98E+05
LCABL_19050	hypothetical protein	yqeK	55,28	22550	8	0,34	6,13E-01	21,527	22,702	20,939	21,088	22,177	20,872	1,39E+06	5,96E+05
LCABL_00070	(phosphodeoxyriboaldolase) (deoxyriboaldolase) (DERA)	deoC	47,86	25375	9	0,29	7,44E-01	20,492	22,476	21,079	21,044	22,522	19,622	1,01E+06	5,90E+05
LCABL_26450	DNA-directed RNA polymerase subunit alpha	rpoA	38,78	34759	6	0,27	6,09E-01	22,396	22,023	22,431	21,811	21,691	22,531	1,08E+06	5,90E+05
LCABL_18580	protein translation Elongation Factor P	efp	14,97	20915	3	-0,45	5,41E-01	19,993	20,441	21,548	21,850	19,930	21,544	6,10E+05	5,85E+05
LCABL_28910	hypothetical protein		45,67	26836	7	0,07	9,15E-01	20,263	22,448	21,213	21,756	21,240	20,708	1,37E+06	5,78E+05
LCABL_27380	CTP synthetase	pyrG	51,5	59781	17	0,91	4,67E-01	22,272	24,047	20,224	20,830	23,408	19,588	1,72E+06	5,78E+05
LCABL_15370	pyruvate dehydrogenase complex, E1	pdhB	44,31	35356	12	0,58	3,52E-01	22,359	22,999	21,431	21,397	22,118	21,544	1,63E+06	5,76E+05
LCABL_16780	aspartate carbamoyltransferase catalytic	pyrB	48,44	35808	10	0,30	7,40E-01	21,063	22,639	19,943	20,678	22,319	19,735	1,25E+06	5,71E+05
LCABL_00090	30S ribosomal protein S6	rpsF	44,9	11572	3	0,30	6,18E-01	21,015	21,780	20,124	20,655	20,435	20,924	1,27E+06	5,62E+05
LCABL_11850	hypothetical protein		10,43	18880	2	0,39	5,19E-01	20,954	20,874	20,777	19,547	20,890	21,012	1,04E+06	5,60E+05
LCABL_04940	dihydroxyacetone kinase subunit DhaK	dak1B	19,17	35947	4	-0,22	7,89E-01	20,915	20,833	21,345	22,155	19,425	22,173	6,16E+05	5,52E+05
LCABL_17260	Glycine--tRNA ligase beta subunit	glyS	41,65	76051	18	0,46	6,45E-01	23,911	21,284	25,050	22,480	22,460	23,923	1,12E+06	5,43E+05
LCABL_08590	hypothetical protein	yslB	28,05	18375	2	0,53	3,70E-01	20,535	20,488	20,264	20,530	20,111	19,045	1,04E+06	5,35E+05
LCABL_10110	UPF0210 protein LCABL_10110		30,59	48222	8	0,03	9,54E-01	21,713	22,113	21,360	21,555	22,154	21,386	8,60E+05	5,32E+05
LCABL_30100	hypothetical protein	yycI	32,45	29681	4	-0,54	3,07E-01	20,669	20,810	21,074	21,554	21,625	20,993	4,96E+05	5,20E+05
LCABL_00020	DNA-directed DNA polymerase III subunit beta	dnaN	21,37	41457	6	-1,42	1,33E-01	20,240	19,098	22,098	22,047	20,894	22,752	2,10E+05	5,19E+05
LCABL_17270	glycyl-tRNA synthetase subunit alpha	glyQ	32,18	30051	5	0,71	4,76E-01	22,077	22,511	19,352	20,228	21,901	19,686	1,48E+06	4,94E+05
LCABL_27010	33 kDa chaperonin	hslO	39,8	31366	7	0,53	4,00E-01	21,853	21,973	20,836	20,655	21,810	20,611	1,06E+06	4,92E+05
LCABL_27880	hypothetical protein		16,08	30372	3	-0,68	3,10E-01	20,361	19,909	21,025	21,734	20,099	21,499	4,09E+05	4,83E+05
LCABL_09330	YfnI	yfnI	22,81	78017	10	0,09	8,84E-01	23,402	22,123	23,413	22,742	22,721	23,220	6,80E+05	4,68E+05
LCABL_19810	ATP-dependent clp protease ATP-binding	clpE	40,69	77221	22	0,06	9,65E-01	19,892	23,635	19,647	19,845	23,798	19,341	7,92E+05	4,62E+05
LCABL_26990	lysyl-tRNA synthetase	lysS	37,15	56537	14	1,51	9,74E-02	22,709	23,791	22,038	20,646	22,940	20,418	1,56E+06	4,56E+05
LCABL_19600	response regulator	rrp1	21,88	25895	4	0,36	6,88E-01	22,427	21,530	19,179	20,786	21,398	19,858	1,10E+06	4,56E+05
LCABL_12330	hypothetical protein	yteA	28,28	32333	6	0,20	7,89E-01	21,296	21,702	19,629	20,341	21,569	20,125	1,01E+06	4,55E+05
LCABL_08270	alpha/beta hydrolase superfamily protein		11,03	31172	2	0,03	9,69E-01	20,777	20,395	22,276	22,052	20,141	21,168	5,75E+05	4,52E+05
LCABL_10730	UPF0042 nucleotide-binding protein		36,95	33609	8	1,03	1,91E-01	22,028	21,668	22,414	21,755	21,793	19,462	7,00E+05	4,51E+05

LCABL_24720	transcription antitermination protein NusG	nusG	59,24	20854	6	-0,07	9,34E-01	18,534	20,789	21,313	20,909	20,740	19,202	5,15E+05	4,50E+05
LCABL_10690	hydrolase of HD superfamily		41,4	25000	5	0,16	8,61E-01	21,511	21,462	19,086	21,006	21,614	18,945	7,40E+05	4,38E+05
LCABL_20280	N-acetylglucosamine-6-phosphate deacetylase	nagA	28,61	42325	6	-0,74	4,66E-01	20,715	19,526	22,363	21,858	19,938	23,021	2,39E+05	4,26E+05
LCABL_16800	bifunctional pyrimidine regulatory protein PyrR uracil phosphoribosyltransferase	pyrR1	28,02	20472	3	-0,80	2,37E-01	19,279	20,211	20,760	21,107	20,084	21,448	4,12E+05	4,25E+05
LCABL_07550	hypothetical protein	yneB	39,46	28463	5	0,56	4,29E-01	20,669	21,792	20,363	19,543	21,380	20,230	1,01E+06	4,24E+05
LCABL_26800	DNA-directed RNA polymerase subunit beta'	rpoC	48,36	136433	43	0,71	5,56E-01	23,510	24,924	21,018	21,556	24,451	21,324	1,15E+06	4,18E+05
LCABL_19300	UDP-N-acetylmuramate--L-alanine ligase	murC	31,42	48843	9	0,86	3,16E-01	21,163	21,462	23,463	22,265	20,140	21,102	9,51E+05	4,08E+05
LCABL_02840	sugar ABC transporter periplasmic protein		17,02	46936	7	-1,11	1,14E-01	21,394	20,358	20,563	22,752	20,955	21,949	2,65E+05	4,05E+05
LCABL_08350	S-adenosylmethionine tRNA ribosyltransferase-	queA	16,85	40417	4	0,45	6,36E-01	21,602	21,385	21,546	22,686	18,909	21,598	5,92E+05	4,04E+05
LCABL_06710	hypothetical protein		16,34	71822	6	-0,87	3,52E-01	21,110	19,786	23,008	22,550	21,082	22,896	3,10E+05	3,99E+05
LCABL_16920	ribonucleotide-diphosphate reductase subunit	nrdE	34,58	81893	15	1,13	2,06E-01	22,483	22,823	22,607	20,120	23,386	21,019	6,88E+05	3,99E+05
LCABL_09500	polysaccharide transport membrane protein		1,65	60623	1	-0,36	5,75E-01	21,794	20,404	21,920	21,620	21,270	22,303	4,32E+05	3,98E+05
LCABL_23000	3-oxoacyl-acyl-carrier-protein] synthase III	fabH	55,62	34718	9	0,41	6,70E-01	20,746	22,497	19,349	19,133	21,456	20,786	1,27E+06	3,87E+05
LCABL_20260	nicotinate phosphoribosyltransferase	nadC	40,66	54686	13	0,45	6,68E-01	22,459	23,306	19,803	21,235	22,829	20,141	1,21E+06	3,86E+05
LCABL_27310	UDP-N-acetylmuramoyl-tripeptide--D-alanyl-D-	murF	47,19	50573	11	0,57	5,48E-01	21,896	22,341	19,667	20,573	22,120	19,508	8,85E+05	3,83E+05
LCABL_02650	branched-chain amino acid ABC transporter	livA	20,91	41746	4	-0,57	5,02E-01	21,902	19,191	21,803	22,039	20,843	21,721	3,63E+05	3,79E+05
LCABL_15800	2 ,3-cyclic-nucleotide 2-phosphodiesterase	ycjM	58,22	56754	11	-0,08	9,08E-01	21,257	22,816	20,439	21,351	22,031	21,377	8,80E+05	3,75E+05
LCABL_13830	ATP synthase subunit c	atpE	38,57	7174	2	-0,95	2,52E-01	19,999	17,012	18,759	19,457	19,232	19,940	4,34E+05	3,73E+05
LCABL_28180	protein recA	recA	23,93	43001	6	0,72	2,78E-01	21,861	21,820	21,968	21,504	21,967	20,003	6,78E+05	3,69E+05
LCABL_07610	hypothetical protein		33,43	39636	8	0,57	6,55E-01	20,468	20,010	22,444	21,824	17,459	21,921	3,90E+05	3,59E+05
LCABL_15420	myo-inositol-1(Or 4)-monophosphatase	suhB	52,47	28133	7	0,85	2,15E-01	20,854	21,841	21,189	19,644	21,475	20,216	8,88E+05	3,59E+05
LCABL_08760	Biotin-[acetyl-CoA-carboxylase] ligase	birA	28,4	26632	4	0,51	4,13E-01	21,060	21,542	20,240	20,491	21,048	19,761	9,24E+05	3,58E+05
LCABL_02440	oligoendopeptidase F1	pepF1	38,4	67252	14	0,70	5,53E-01	22,406	23,384	19,122	20,507	22,368	19,945	1,34E+06	3,57E+05
LCABL_07440	tagatose 1,6-diphosphate aldolase	lacD2	41,14	36393	5	-0,82	1,74E-01	21,587	21,172	20,284	21,745	21,579	22,188	4,92E+05	3,56E+05
LCABL_27220	YciB protein	yciB	11,11	26536	2	-0,37	5,36E-01	20,651	19,278	20,621	20,424	20,531	20,691	3,67E+05	3,54E+05
LCABL_15660	glutamine ABC transporter ATP-binding protein	glnQ3	54,03	27311	8	0,17	8,20E-01	20,669	21,992	19,815	20,915	21,224	19,842	7,17E+05	3,53E+05
LCABL_27060	hypothetical protein		13,89	90363	8	-0,12	8,58E-01	22,450	20,872	22,737	21,765	21,717	22,951	4,40E+05	3,51E+05
LCABL_13790	translation factor SUA5		31,53	34859	4	0,59	4,11E-01	21,391	21,610	19,537	20,130	20,832	19,792	9,82E+05	3,40E+05
LCABL_25180	Probable potassium transport system protein	kup	1,17	76719	1	-0,19	7,18E-01	21,740	21,171	21,964	22,018	21,516	21,911	4,14E+05	3,35E+05
LCABL_03550	esterase C	estC	37,45	29012	5	0,35	6,92E-01	20,437	22,191	19,525	20,631	21,396	19,070	8,54E+05	3,26E+05
LCABL_05320	cell envelope-associated proteinase PrtR	prtR	8,29	236397	12	-0,08	9,11E-01	24,194	22,171	24,054	23,504	22,992	24,160	4,35E+05	3,22E+05
LCABL_15200	cysteine desulfurase	csd2	8,01	41842	3	-0,40	7,31E-01	17,513	20,472	22,099	20,551	19,265	21,474	5,62E+05	3,18E+05
LCABL_28060	Aldo/keto reductase		30,84	37050	6	-0,68	2,78E-01	20,230	20,796	19,460	21,480	20,678	20,370	3,62E+05	3,15E+05
LCABL_12250	lipid kinase		31,98	37318	9	-0,21	8,27E-01	21,511	22,064	18,559	20,554	21,520	20,676	9,31E+05	3,13E+05
LCABL_06340	(phosphodeoxyriboaldolase) (deoxyriboaldolase) (DERA)	deoC	18,75	26425	3	-0,09	9,02E-01	20,649	21,034	19,250	20,895	20,979	19,326	4,85E+05	3,09E+05
LCABL_10760	MarR family transcriptional regulator	ohrR	33,11	17336	3	-0,18	7,96E-01	19,775	20,209	19,085	18,944	19,613	21,055	3,90E+05	3,02E+05
LCABL_13870	ATP synthase gamma chain	atpG	30,52	33709	4	0,25	7,32E-01	20,569	21,428	19,661	20,726	21,001	19,182	5,66E+05	2,97E+05
LCABL_16110	1| hypothetical protein	ORF	17,08	30603	3	-0,23	8,22E-01	20,449	19,032	18,447	17,276	20,598	20,751	2,86E+05	2,96E+05
LCABL_30630	galactoside O-acetyltransferase	thgA3	19,07	23811	2	-0,51	4,71E-01	18,140	19,228	19,611	20,605	18,752	19,166	3,01E+05	2,95E+05
LCABL_30330	EIIC	manM	6,27	27738	1	0,55	5,65E-01	20,371	18,738	21,127	20,455	17,761	20,378	3,18E+05	2,92E+05
LCABL_31030	Mannitol-1-phosphate 5-dehydrogenase	mtlD	28,2	42453	6	-0,11	9,03E-01	20,978	20,434	22,780	21,413	20,273	22,824	2,79E+05	2,84E+05
LCABL_01990	16S rRNA methyltransferase GidB	gidB	33,2	26639	7	0,88	3,50E-01	20,037	22,071	20,047	20,115	21,172	18,214	8,89E+05	2,84E+05
LCABL_16280	ATP-dependent protease subunit HslV	hslV	16,67	18743	3	0,12	8,13E-01	19,379	20,148	19,378	19,406	19,657	19,473	5,23E+05	2,69E+05
LCABL_15570	BS_ysoA related protein with TPR repeats		41,91	33497	6	0,22	7,99E-01	20,445	21,855	19,181	19,225	21,364	20,239	7,39E+05	2,65E+05
LCABL_16950	oligoendopeptidase O	pepO	23,34	71505	13	0,02	9,84E-01	21,473	19,777	24,075	21,063	20,697	23,494	3,96E+05	2,59E+05
LCABL_02130	aspartate racemase	racD	31,2	28260	4	0,59	3,81E-01	19,765	21,271	19,552	19,069	20,207	19,529	8,04E+05	2,56E+05
LCABL_19560	beta-lactamase-like		37,55	26876	5	0,67	3,01E-01	20,370	21,209	19,735	20,324	19,851	19,142	7,57E+05	2,54E+05
LCABL_22120	ClpL	clpL	26,4	78536	11	1,53	2,13E-01	24,738	20,612	24,811	20,754	22,386	22,434	1,06E+06	2,48E+05
LCABL_11810	YbbR	ybbR	21,88	38199	5	-0,73	5,79E-01	19,475	17,655	22,238	21,412	18,411	21,742	2,17E+05	2,44E+05
LCABL_23900	hypothetical protein		10,49	18026	2	-0,41	5,41E-01	19,357	18,593	19,224	19,497	18,439	20,463	2,66E+05	2,41E+05
LCABL_08720	bifunctional glycosyltransferase/transpeptidase	pbp1B	18	68932	6	-0,84	3,93E-01	21,106	20,018	18,544	18,952	21,555	21,670	2,01E+05	2,39E+05
LCABL_04630	flavodoxin		32,21	23491	2	0,22	7,57E-01	20,759	19,069	20,658	19,001	20,627	20,195	3,10E+05	2,38E+05
LCABL_20650	acetolactate synthase	als	37,63	60354	8	-0,24	7,25E-01	21,330	22,075	19,839	21,235	21,446	21,277	5,75E+05	2,36E+05
LCABL_18400	Methionyl-tRNA formyltransferase	fmt	27,99	34370	4	-0,04	9,44E-01	20,768	21,592	20,251	21,005	21,353	20,378	5,76E+05	2,31E+05
LCABL_19110	lactoylglutathione lyase related lyase		32,3	36510	6	0,61	3,85E-01	19,881	21,107	19,704	18,739	20,571	19,539	6,79E+05	2,30E+05
LCABL_18900	phenylalanyl-tRNA synthetase subunit beta	pheT	22,91	87704	9	-0,11	8,97E-01	21,440	22,301	19,131	20,595	21,826	20,790	4,98E+05	2,20E+05
LCABL_09430	S-adenosylmethionine synthase	metK	14,97	43000	5	-0,04	9,52E-01	20,601	21,190	19,361	20,783	20,459	20,025	4,41E+05	2,20E+05
LCABL_08190	acetyltransferase	wecD	68,79	19203	5	0,42	5,60E-01	19,342	21,007	19,300	19,116	20,362	18,926	5,19E+05	2,19E+05
LCABL_03660	beta-lactamase class A		11,07	30333	2	-0,34	6,48E-01	19,434	18,921	20,726	20,109	19,036	20,945	2,40E+05	2,19E+05
LCABL_07180	YkpA protein	ykpA	33,88	61537	8	0,77	2,33E-01	21,273	22,231	21,095	19,942	21,208	21,134	6,79E+05	2,18E+05
LCABL_17420	Aspartate--tRNA ligase	aspS	45,09	67554	15	2,48	3,46E-03	22,872	24,520	24,342	20,954	21,596	21,741	2,27E+06	2,18E+05
LCABL_08260	hypothetical protein		14,29	33349	3	-0,30	6,20E-01	20,848	19,434	19,485	20,598	19,961	20,109	2,70E+05	2,15E+05
LCABL_18520	Farnesyl-diphosphate synthase	ispA	15	29804	2	-0,13	8,87E-01	20,391	20,105	17,599	20,234	18,074	20,188	4,54E+05	2,14E+05
LCABL_10930	hypothetical protein	ORF28	32,93	18605	4	0,50	5,05E-01	19,763	21,754	20,014	20,608	19,058	20,361	7,75E+05	2,08E+05
LCABL_03250	RbsB (ribose ABC transporter) (ribose-binding	rbsB	24,45	33282	3	-0,01	9,86E-01	20,356	19,765	21,333	20,546	20,010	20,930	2,83E+05	2,05E+05
LCABL_13370	Major tail protein	mtp	6,03	21877	1	-0,10	8,72E-01	20,016	18,402	19,255	19,005	19,328	19,631	2,69E+05	2,01E+05
LCABL_19210	Threonine--tRNA ligase	thrS	37,6	74588	17	1,63	2,67E-01	23,672	20,122	24,977	21,566	19,178	23,151	8,70E+05	2,00E+05
LCABL_17920	prolyl-tRNA synthetase	proS	69,08	64859	29	3,60	5,33E-03	23,141	24,202	24,123	21,790	20,931	17,930	2,31E+06	1,98E+05
LCABL_20350	hypothetical protein		7,51	84110	4	-0,50	4,32E-01	21,575	19,951	21,435	21,621	21,094	21,750	1,93E+05	1,97E+05
LCABL_30320	EIID	mann	21,78	33473	4	0,60	3,97E-01	20,352	20,479	20,572	20,401	18,480	20,714	3,41E+05	1,91E+05
LCABL_27150	peptidyl-tRNA hydrolase	pth	16,22	20820	3	0,07	9,32E-01	19,928	20,380	17,892	19,709	19,878	18,409	3,99E+05	1,90E+05
LCABL_19840	Peptide chain release factor 3	prfC	28,63	59493	9	1,01	2,52E-01	20,654	21,906	20,052	18,780	21,450	19,340	4,82E+05	1,87E+05
LCABL_22300	hypothetical protein		1,67	47601	1	-0,25	6,89E-01	20,362	19,280	20,908	20,266	20,022	21,021	2,15E+05	1,87E+05

LCABL_11390	exoribonuclease R, RNase II family	rnr	16,73	88952	8	-1,01	2,15E-01	19,203	22,012	20,534	21,232	21,478	22,068	2,75E+05	1,86E+05
LCABL_11570	sugar ABC transporter ATPase	malK	17,27	39001	3	0,43	4,61E-01	19,860	20,194	19,737	20,071	19,708	18,732	3,61E+05	1,85E+05
LCABL_18190	Signal recognition particle-docking protein FtsY	ftsY	35,65	36154	7	0,82	2,99E-01	20,938	22,191	20,154	19,859	21,400	19,574	5,50E+05	1,81E+05
LCABL_10240	GMP reductase	guaC	16,72	36504	3	0,13	8,40E-01	20,530	20,473	19,325	20,102	20,720	19,111	2,78E+05	1,75E+05
LCABL_11680	reductase (UDP-N-acetylmuramate dehydrogenase)	murB	18,27	32237	4	0,43	6,14E-01	21,248	20,968	18,702	19,076	20,877	19,683	4,61E+05	1,74E+05
LCABL_02300	hypothetical protein		5,38	53821	2	-0,65	4,58E-01	18,945	19,257	21,214	20,434	19,242	21,696	1,59E+05	1,72E+05
LCABL_22340	Wze	wze	25,4	27606	5	0,13	8,73E-01	20,097	20,916	18,420	19,687	20,468	18,903	3,66E+05	1,67E+05
LCABL_18270	peptide ABC transporter ATP-binding protein	oppD	19,12	37944	4	0,72	2,98E-01	19,832	21,372	20,758	19,501	19,468	20,847	5,74E+05	1,67E+05
LCABL_24880	Cysteine--tRNA ligase	cysS	23,72	53502	8	0,86	3,45E-01	20,681	21,967	19,871	18,503	21,435	19,995	4,52E+05	1,64E+05
LCABL_14780	cysteine desulfurase / Selenocysteine lyase	nifS	17,92	41895	5	0,83	2,64E-01	21,085	20,550	22,290	20,067	19,839	21,527	2,98E+05	1,62E+05
LCABL_11530	exonuclease of the beta-lactamase fold	YSH1	21,2	46711	6	0,23	7,99E-01	17,949	20,427	21,440	20,194	19,705	19,222	3,56E+05	1,61E+05
LCABL_27970	glycine betaine/carnitine/choline ABC transporter substrate-binding and permease	choS	8,63	55523	3	-0,33	6,90E-01	19,400	19,778	21,859	20,544	19,775	21,720	1,93E+05	1,59E+05
LCABL_07300	Galactokinase	galK	11,34	42475	3	-0,32	7,25E-01	18,356	19,314	21,382	20,048	18,884	21,094	2,07E+05	1,58E+05
LCABL_26830	ATP-dependent Clp protease ATP-binding	clpC	27,19	91748	13	0,53	7,40E-01	20,518	22,872	17,613	19,757	22,376	17,291	4,12E+05	1,57E+05
LCABL_10860	anti-repressor	orf6	17,9	29726	1	0,65	2,70E-01	20,541	20,395	19,877	18,928	20,108	19,836	3,64E+05	1,56E+05
LCABL_10550	phosphate transporter protein	phoU	24	25875	3	0,63	3,91E-01	19,964	20,809	18,854	18,704	20,233	18,789	4,13E+05	1,54E+05
LCABL_15290	mRNA degradation ribonuclease J1/J2, metallo-beta-lactamase superfamily enzyme		21,07	61597	11	0,37	7,33E-01	19,109	22,028	18,832	18,970	21,402	18,480	4,17E+05	1,53E+05
LCABL_10420	Protein translocase subunit SecA	secA	25,41	89367	13	0,84	4,29E-01	21,720	22,601	19,467	20,167	22,093	19,005	4,16E+05	1,49E+05
LCABL_15810	hypothetical protein		7,26	31751	3	-0,71	1,92E-01	19,771	19,400	19,261	20,441	19,797	20,326	1,43E+05	1,47E+05
LCABL_16120	hypothetical protein		5,26	23444	1	-0,23	7,19E-01	19,050	18,702	20,231	19,650	18,859	20,174	1,75E+05	1,45E+05
LCABL_17390	endonuclease IV	nfo	39,37	31515	7	1,14	1,94E-01	19,683	21,774	19,501	18,396	20,362	18,790	6,05E+05	1,41E+05
LCABL_01600	acetate kinase	ackA	11,36	43235	3	0,45	6,43E-01	20,894	18,536	21,451	18,918	19,311	21,309	2,41E+05	1,37E+05
LCABL_21460	peptidase M42 family	ysdC	7,2	39832	2	-0,69	5,13E-01	19,582	16,990	20,190	19,520	18,220	21,079	1,12E+05	1,34E+05
LCABL_23860	cytochrome D ubiquinol oxidase subunit I	cydA	1,48	53521	1	0,03	9,58E-01	19,506	19,061	19,991	19,022	19,372	20,074	1,91E+05	1,33E+05
LCABL_20250	serine-type D-Ala-D-Ala carboxypeptidase	pbpX2	19,9	44161	6	0,12	9,00E-01	19,961	18,951	21,601	20,443	18,465	21,251	1,81E+05	1,30E+05
LCABL_21970	Tyrosyl-tRNA synthetase (tyrosine--tRNA ligase)	tyrS	24,06	47764	9	2,00	3,14E-02	21,005	21,254	22,782	20,961	19,509	18,577	5,50E+05	1,28E+05
LCABL_15690	fructose-specific phosphotransferase system,	fruA	5,79	69974	2	-0,03	9,60E-01	19,703	20,093	20,053	20,112	19,377	20,441	1,96E+05	1,18E+05
LCABL_20020	pyruvate oxidase	poxL	20,55	63962	6	1,03	1,18E-01	20,940	21,355	20,608	20,743	19,965	19,091	3,27E+05	1,17E+05
LCABL_16750	Carbamoyl-phosphate synthase large chain	carB	25,57	115985	16	0,18	8,75E-01	20,755	22,502	18,537	19,664	22,217	19,368	2,76E+05	1,16E+05
LCABL_28900	hypothetical protein	YPO2511	2,92	42314	1	-0,26	7,51E-01	20,451	18,317	20,935	19,713	19,655	21,121	1,24E+05	1,08E+05
LCABL_16960	Lipoate-protein ligase	lplA1	15,73	38457	4	0,28	6,71E-01	20,089	20,764	18,744	19,470	19,549	19,750	2,91E+05	1,04E+05
LCABL_13850	ATP synthase subunit delta	atpH	33,7	19361	2	-0,84	1,80E-01	17,724	19,140	19,086	19,474	19,752	19,257	2,08E+05	9,96E+04
LCABL_29780	endonuclease/exonuclease/phosphatase		15,21	39903	3	0,35	6,53E-01	20,701	18,842	19,168	20,396	18,410	18,867	1,71E+05	9,92E+04
LCABL_27610	deoxyribonuclease	tatD	10,81	29245	3	1,28	3,18E-02	20,612	20,348	20,242	19,057	19,513	18,790	2,89E+05	9,78E+04
LCABL_01130	Adenylosuccinate synthetase	purA	19,26	47422	6	1,65	8,67E-02	20,286	21,433	22,340	20,625	18,040	20,446	4,45E+05	9,63E+04
LCABL_02750	phosphopentomutase	deoB	12,12	43110	3	0,48	5,93E-01	19,988	18,897	20,793	18,435	18,628	21,182	1,52E+05	9,35E+04
LCABL_22890	Cyclopropane-fatty-acyl-phospholipid synthase	cfa	21,63	45242	4	1,82	1,46E-01	20,537	19,631	22,141	19,038	16,675	21,151	2,35E+05	8,95E+04
LCABL_28780	hypothetical protein		13,83	48583	3	-0,41	5,02E-01	20,599	19,411	19,490	20,319	19,677	20,722	1,32E+05	7,77E+04
LCABL_00600	MMPL precursor		7,8	116912	3	-2,07	2,88E-02	20,003	18,422	17,933	21,181	19,584	21,797	4,48E+04	6,59E+04
LCABL_21080	oligopeptide transport ATP-binding protein	oppF	14,38	32239	3	1,13	7,45E-02	18,733	20,001	19,505	18,000	18,694	18,161	2,62E+05	5,84E+04
LCABL_20410	phosphoenolpyruvate carboxykinase (ATP)	pck	22,88	60985	8	0,55	4,90E-01	19,730	21,512	18,732	19,169	19,984	19,184	2,65E+05	5,74E+04
LCABL_12830	phosphoglycerol transferase		5,46	83931	2	0,67	3,94E-01	20,400	18,978	20,553	18,445	18,875	20,612	8,09E+04	5,25E+04
LCABL_15030	cell division protein, FtsQ	ftsQ	13,59	32549	3	0,27	5,90E-01	18,756	19,320	18,940	18,615	18,925	18,655	1,25E+05	5,22E+04
LCABL_18120	Ribosome maturation factor rimM	rimM	10,47	19029	1	-0,10	8,62E-01	18,094	18,099	18,469	18,375	17,603	18,979	1,05E+05	4,84E+04
LCABL_15360	pyruvate dehydrogenase complex, E1	pdhA	3,51	40813	1	0,20	8,22E-01	18,904	16,570	19,463	18,040	17,218	19,081	3,86E+04	4,17E+04
LCABL_17430	Histidine--tRNA ligase	hisS	23,89	47565	3	0,20	7,35E-01	19,500	19,704	20,831	20,211	19,627	19,600	1,54E+05	4,07E+04
LCABL_15610	ATP-dependent protease ATP-binding protein	clpX	8,89	45753	1	0,20	8,24E-01	17,616	18,376	20,291	18,040	17,703	19,942	6,74E+04	3,84E+04
LCABL_19450	bifunctional	pbp2A	6,47	74852	2	-0,02	9,73E-01	18,734	18,049	18,586	19,335	17,732	18,362	3,45E+04	2,63E+04
LCABL_23430	asparagine synthetase	asnH	14,35	73066	7	3,17	6,88E-03	20,246	19,657	23,050	17,486	17,512	18,442	2,20E+05	8,93E+03
LCABL_20300	peptidase, S9 family	yuxL	8,36	73595	4	-1,64	7,76E-02	16,704	18,539	17,937	20,075	17,776	20,248	3,51E+04	8,52E+03
